# Reconfigurable All-Nitride Magneto-Ionics

**DOI:** 10.1021/acsnano.5c04013

**Published:** 2025-05-07

**Authors:** Zhijie Chen, Christopher J. Jensen, Chen Liu, Yijing Liu, Christy J. Kinane, Andrew John Caruana, Alexander J. Grutter, Julie A. Borchers, Xixiang Zhang, Kai Liu

**Affiliations:** † Physics Department, 8368 Georgetown University, Washington, District of Columbia 20057, United States; ‡ NIST Center for Neutron Research, 10833 National Institute of Standards and Technology, Gaithersburg, Maryland 20899, United States; § 127355 King Abdullah University of Science & Technology, Thuwal 23955-6900, Saudi Arabia; ∥ ISIS Neutron Facility, STFC Rutherford Appleton Laboratory, Chilton OX11 0QX, Oxfordshire, U.K.

**Keywords:** voltage control of magnetism, magneto-ionics, exchange bias, manganese nitride, antiperovskite, Mn_4_N

## Abstract

The rapid advancement of generative artificial intelligence has significantly increased the demand for both energy and data storage. Magneto-ionics, which utilizes ionic motion to control magnetism, often driven by an electric field in heterostructures, has gained significant attention for its potential to enable energy-efficient modulation of magnetic properties with large effects. This study proposes a CMOS-compatible solid-state magneto-ionic system composed of all-Mn-nitrides and demonstrates that nitrogen ionic motion can induce reversible phase transitions between ferrimagnetic and antiferromagnetic Mn nitrides. This magnetic phase transition is manifested in dramatic changes in the resultant exchange bias effect, which can be increased by over an order of magnitude when more nitrogen is introduced into the nitrides during deposition and subsequently reduced by over 70% when nitrogen is taken out of the nitrides through post-annealing. Additionally, voltage-induced nitrogen ionic motion can lead to reversible changes in saturation magnetization and the exchange bias effect by 23% and 0.1 T (16%) at 5 K, respectively. These findings highlight the characteristics of this all-Mn-nitride system as an industrially viable and environmentally sustainable platform, offering tunable magnetic properties and energy-efficient operation and potential for magnetic field immunity.

## Graphical abstract

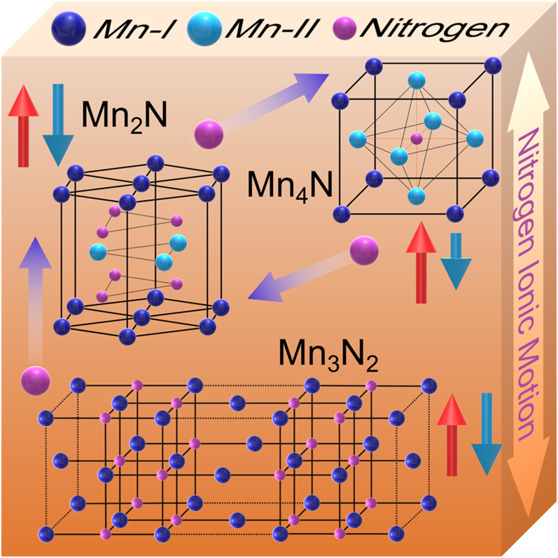

The emergence of generative artificial intelligence, which are machine learning models that create new content by learning and emulating patterns from existing data sets, has led to significant advancements and widespread application of large language models like ChatGPT. However, training and maintaining these models require substantial computational resources, leading to a considerable increase in power consumption in the information and communications technology sector, projected to consume all energies produced globally by the 2040s based on current technology.^1^ Additionally, the storage demands for vast amounts of data have resulted in a surge of data centers, further exacerbating energy requirements.^2,3^ This unsustainable energy thirst must be addressed with new paradigms of computing with fundamentally different operating principles. One promising solution lies in the voltage control of magnetism (VCM),^4,5^ which significantly reduces energy consumption by minimizing Joule heating while maintaining compatibility with the semiconductor industry. To this end, there has been a surge of interest in multiferroic and magnetoelectric materials,^6^ yet significant challenges still exist, in terms of nonvolatility, limited tunability, and scalability.^4,7^

In recent years, magneto-ionics has emerged as a promising method to tailor magnetic properties through controlled ionic motion,^8–25^ with attractive features such as large effect sizes, energy efficiency, unconventional functionalities, and potential applications in neuromorphic computing.^26,27^ Several methods have been developed to induce the ionic motion, including electrolyte gating,^8,15^ solid-state gating,^11,23,25^ chemisorption,^17,20^ and redox reactions.^12–14^ These approaches allow for the regulation of magnetic properties such as saturation magnetization,^28^ magnetic anisotropy,^9,10^ exchange bias,^12,23,25,29^ Dzyaloshinskii-Moriya interaction,^17^ and spin textures.^24^ Moreover, various ionic species such as oxygen,^9–14^ hydrogen,^16,20,22,24,29–31^ nitrogen,^18,21,25,34^ hydroxide,^28^ and lithium^32^ have been investigated for their effectiveness in magneto-ionic applications. Recent studies have highlighted the advantages of nitrogen-based magneto-ionics, which exhibit faster ionic motion and enhanced reversibility, making them particularly promising for future applications.^18,21,25,33,34^ To date, essentially all magneto-ionic systems have been heterogeneous, consisting of dissimilar materials of a functional layer whose magnetic characteristics are being modulated and another electrolyte layer, in either solid or liquid form.

In this study, we introduce a CMOS-compatible all-Mn-nitride magneto-ionic system whose magnetic phases can be dynamically tuned through nitrogen ion migration. The Mn–N phase diagram comprises both antiferromagnetic (AF) and ferrimagnetic (FiM) phases, namely, θ-MnN (AF),^35^ η-Mn_3_N_2_ (AF),^36,37^ ζ-Mn_2_N (AF),^38^ and ε-Mn_4_N (FiM).^39^ Among these, Mn_4_N stands out as the sole FiM Mn nitride, attracting significant attention in recent years as a rare-earth-free and heavy-metal-free material for sustainable spintronics applications.^39–46^ Our previous study shows that the Mn_4_N thin films could be synthesized via the ionically driven synthesis method by depositing Mn onto a Mn_3_N_2_ base layer.^47^ Here, we demonstrate dynamic and magneto-ionic control of magnetic phases in this all-Mn-nitride platform. The nitrogen content in this all-nitride system can be continuously varied to exhibit AF, a mixture of AF and FiM, or FiM phases and manifested in the emergence of a large and tunable exchange bias effect. Notably, the exchange bias can be enhanced by more than an order of magnitude through increasing nitrogen partial pressure during deposition and subsequently reduced by 70% when nitrogen is driven out of the nitrides through post-annealing. Additionally, nitrogen ionic motions induced by room-temperature solid-state voltage application can lead to reversible changes in exchange bias by up to 0.1 T. These changes are mainly attributed to the phase transformation between the AF Mn_2_N and FiM Mn_4_N induced by the nitrogen ionic motion, with higher Mn_2_N content leading to larger exchange bias and vice versa. The nitrogen motions are also confirmed through X-ray diffraction, magnetometry, and polarized neutron reflectivity studies. Our findings highlight the exceptional functionality and versatility of the all-nitride system, where magnetic phases can be toggled between AF and FiM states through deposition, thermal treatment, and voltage biasing. These capabilities position the all-nitride system as a promising platform for energy-efficient and sustainable spintronic applications with the added potential for magnetic field immunity in data storage and memory.

## Results

### Film Structural and Material Characterizations

Mn_3_N_2_ is used as a seed layer that provides the crystalline texture and a source of nitrogen needed for Mn_4_N growth (Figure 1a,b). Mn_3_N_2_ is an AF with a high Néel temperature (*T*_N_ ≈ 925 K),^37,48^ and it can lose nitrogen via thermal and/or chemical interactions and transform into the next stable Mn nitride phase, Mn_2_N, which has a hexagonal structure and is also an AF with *T*_N_ ≈ 300 K (Figure 1c).^35,38^ To start, 20 nm of the Mn_3_N_2_ seed layer is deposited onto Si substrate with a 285 nm SiO_2_ layer at 450 °C. X-ray diffraction (XRD) scans have confirmed the successful growth of this seed layer (Figure S1). Subsequently, 40 nm of pure Mn is deposited onto this seed layer at the same substrate temperature. As shown in Figure 1d, a Mn_4_N single-phase film can be formed, where one prominent peak around *2*θ ≈ 47.1° is observed. The formation of Mn_4_N is due to the chemical reaction between the Mn_3_N_2_ seed layer and the added Mn at high temperature, as explained in our previous study.^47^

**Figure 1:**
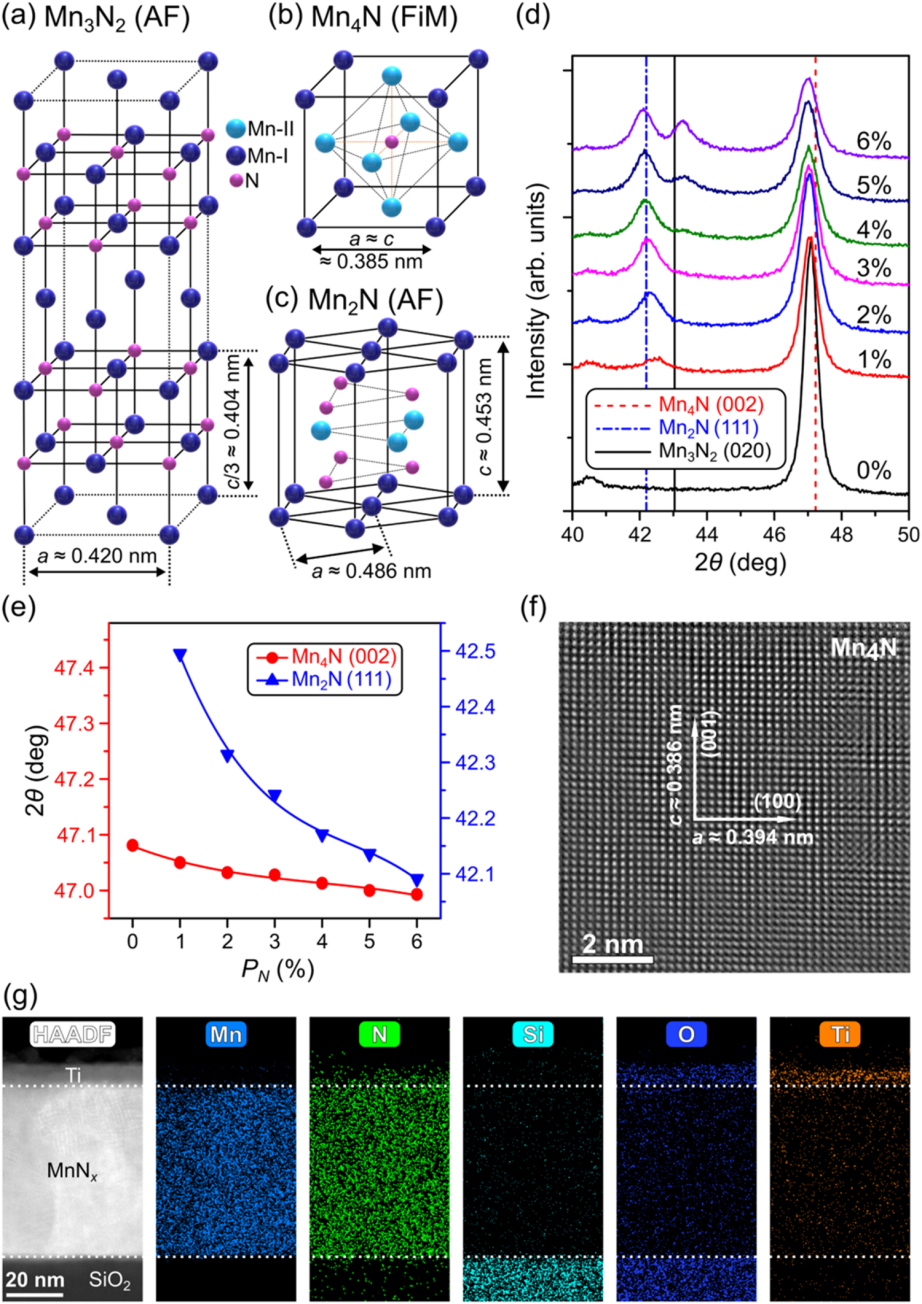
Structural characterizations for the pressure series. Schematics showing the lattice structures of (a) Mn_3_N_2_, (b) Mn_4_N, and (c) Mn_2_N. (d) XRD 2θ-ω scans showing the phase evolution of the pressure series samples with Mn_3_N_2_ (20 nm)/Mn (40 nm) as the nominal layer structures fabricated with different *P*_N_, where *P*_N_ is the nitrogen partial pressure during the Mn deposition. Vertical lines show the expected peak locations of Mn_4_N (002) (red), Mn_2_N (111) (blue), and Mn_3_N_2_ (020) (black). X-ray source is Cu K_α_ with a 0.154 nm wavelength. (e) Trends showing Mn_4_N (002) (red) and Mn_2_N (111) (blue) peak location (extracted from (d)) variations as *P*_N_ changes. Solid lines are a guide to the eye. Error bars are smaller than the graph point size. (f) High-resolution STEM image and (g) EDX elemental maps on a *P*_N_ = 0% sample.

We then varied the nitrogen partial pressure (*P*_N_) during the deposition of the Mn layer while keeping all other deposition parameters the same, equivalent to adding an additional nitrogen source, compared to the initial Mn_4_N single phase grown at *P*_N_ = 0%. These samples are termed the pressure series. As *P*_N_ increases, a peak near 2θ ≈ 42.2° emerges and its integrated intensity grows larger (Figure 1d). This peak is the (111) Bragg peak from the ζ-phase Mn_2_N, which has thermal stability and nitrogen content between η-Mn_3_N_2_ and ε-Mn_4_N.^35,49,50^ Eventually, as *P*_N_ reaches 4%, the Mn_3_N_2_ (020) peak emerges and becomes more prominent, indicating that some of the Mn_3_N_2_ phase in the seed layer persists. These results show that by increasing *P*_N_ during Mn deposition, the layers can be transformed continuously from the Mn_4_N phase into Mn_4_N/Mn_2_N, and then to Mn_4_N/Mn_2_N/Mn_3_N_2_ mixed phases. Grazing incidence XRD also shows the same phase transformations as Figure 1d, along with full range 2θ-ω scans (Figure S1). The nitrogen concentration and phase evolution in the nitride films can be clearly seen from the XRD patterns. Mn nitride lattice parameters are known to be susceptible to the nitrogen concentration, where the interstitial nitrogen usually causes the lattice to expand and nitrogen vacancies would do the opposite.^49^ As shown in Figure 1e, Mn_4_N and Mn_2_N peaks both shift to lower angles as *P*_N_ increases, consistent with the fact that more nitrogen is being incorporated into their lattices.

To study the crystalline quality, scanning transmission electron microscopy (STEM) was performed on a *P*_N_ = 0% sample. Highly ordered cubic Mn_4_N crystal can be clearly seen in Figure 1f. We also identified a small in-plane tensile strain from the STEM image, where the in-plane lattice constant (0.394 nm) is larger than the out-of-plane lattice constant (0.386 nm). This also agrees with the observed out-of-plane Mn_4_N (002) peak located at 2θ ≈ 47.1°. The tetragonal lattice distortion is also believed to be the origin of the PMA in Mn_4_N thin films.^41,42^ Note that the STEM image in Figure 1f specifically represents an individual crystallite. However, grain boundaries and limited long-range crystalline order persist throughout the film. As confirmed by the XRD results (Figure S1), the films exhibit a preferred out-of-plane orientation along the (001) direction, but lack in-plane alignment, which is typical for textured films grown on amorphous substrate. High-angle annular dark-field (HAADF) STEM images and energy-dispersive X-ray spectroscopy (EDX) elemental maps of the sample cross-section are obtained from the same *P*_N_ = 0% sample (Figure 1g). These images again demonstrated high-quality films with homogeneous distribution of Mn and N inside the Mn nitride layer, while nitrogen tends to move into the capping layer due to its high mobility and Ta’s affinity for nitrogen.

### Tuning Magnetic Properties through Nitrogen Partial Pressure

We then investigate how the magnetic properties, particularly the exchange bias (EB), evolve with increasing nitrogen content in these pressure series samples. EB refers to the pinning of an FM (or FiM) layer by an adjacent AF layer, manifested in a shifted hysteresis loop.^51,52^ This effect arises from uncompensated AF spins at the interface, which induce a unidirectional anisotropy in the FM (or FiM) layer after cooling through the AF’s Néel temperature in an applied magnetic field. Hysteresis loops were measured at 5 K after cooling from room temperature with an out-of-plane (OP) and in-plane (IP) positive 2 T magnetic field, respectively. A clear shift of the hysteresis loops to the negative field direction is observed in both the OP and IP loops (Figure 2a,b, respectively). Moreover, the OP loops are wider and more square than the IP loop, consistent with the PMA reported in Mn_4_N films.^40–43^ A close examination reveals that many of the hysteresis loops exhibit a pronounced asymmetry, where the descending-field branch is sharper than the ascending-field branch. This asymmetry arises from different magnetization reversal mechanisms along the ascending and descending branches, often manifestation of the competition between the unidirectional exchange anisotropy and other crystalline anisotropy or the presence of local incomplete domain walls.^51–55^

**Figure 2:**
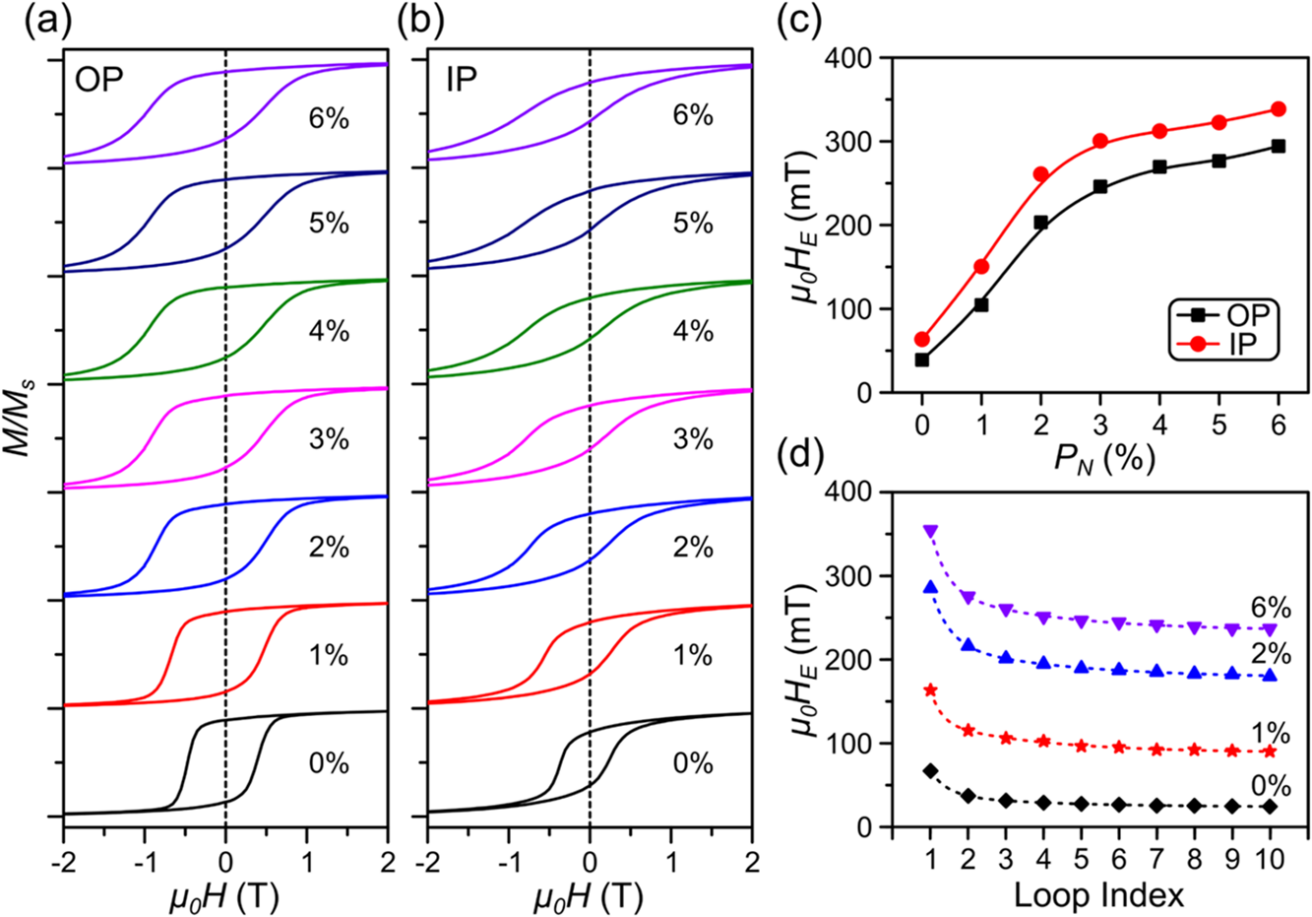
Hysteresis loops and training effects for the pressure series. Hysteresis loops measured at 5 K with (a) out-of-plane (OP) and (b) in-plane (IP) fields after +2 T field cooling for samples in the pressure series. The cooling field and measurement field have the same orientation. Loops are shifted vertically for clarity. (c) Dependence of exchange fields (μ_0_ *H*_E_) on the nitrogen partial pressure (*P*_N_). (d) Training effect for selected samples extracted from 10 consecutive hysteresis loops at 5 K after field cooling from 380 K in +2 T IP field. Solid lines are a guide to the eyes, and dashed lines in (d) are fits to eq 1. Error bars in (c, d) are smaller than the graph point size.

As *P*_N_ increases from 0 to 6%, both the IP and OP exchange field (μ_0_ *H*_E_) increase monotonically (Figure 2c). Notably, there is a rapid ascent from 0 to 2%, followed by a more gradual rise from 3 to 6%. This trend can be directly associated with the phase transitions identified in the XRD data (Figure 1d), which show that the AF Mn_2_N peak evolves from unnoticeable (*P*_N_ = 0%) to prominent (*P*_N_ = 2%). This peak then stays relatively constant from 3 to 6% while another Mn_3_N_2_ peak emerges and grows larger. The EB is mainly attributed to the interaction between FiM Mn_4_N and AF Mn_2_N.^47^ Mn_3_N_2_, despite being AF as well, is not expected to contribute to EB significantly, as the field cooling was done well below its rather high *T*_N_ (≈925 K).

We also studied the EB field training effect. Samples from the pressure series were initially field cooled from 380 to 5 K in a 2 T IP field before ten consecutive hysteresis loops were completed. The exchange field extracted from each of the ten loops exhibits an exponential decay as loop number increases (Figure 2d), which is typical for EB systems. It can be further fitted with the following model considering both the rotatable and frozen spins near the interfaces^56–58^

1 $$\mu_{0}H_{E}^{n}=\mu_{0}H_{E}^{\infty }+A_{F}\;\exp\left(-\frac{n}{P_{F}}\right)+A_{R}\;\exp\left(-\frac{n}{P_{R}}\right)$$

where *n* is the loop number, μ_0_ *H*_E_^*n*^ and μ_0_ *H*_E_^∞^ are the exchange field of the *n*th loop and the equilibrium exchange field, respectively, *A*_F_ and *A*_R_ are parameters with magnetic field units that are related to the frozen and rotatable spins, respectively, and *P*_F_ and *P*_R_, on the other hand, are dimensionless parameters that resemble relaxation times for the frozen and rotatable spins, respectively. The fitted curves are shown as dashed lines in Figure 2d. The remarkable tunability of EB is manifested in the 10-fold increase of μ_0_ *H*_E_^∞^, from μ_0_ *H*_E_^∞^ ≈ 23 mT for the *P*_N_ = 0% sample to 234 mT for the *P*_N_ = 6% sample. The fitting parameters can be found in Table S1 (Supporting Information).

To further elucidate the origin of the EB effect, we have studied the temperature and cooling field dependence of the EB, included in Figure S2. An intriguing spin-glass-like phase is found in this Mn nitride system, stemming from the competing exchange interactions in the mixed AF and FiM phases.^58–65^ We have also investigated the room-temperature magnetization reversal behavior using the first-order reversal curve (FORC) method^66–69^ and included the results in Figure S3. FORC is known as a powerful characterization technique that can disentangle different magnetic interactions and provide insights that are not attainable with conventional major hysteresis loops. FORC confirmed the existence of the single Mn_4_N phase with PMA in the *P*_N_ = 0% sample, and it revealed another softer magnetic phase with smaller coercivities when *P*_N_ increases, which likely resulted from the Mn_4_N phase fragmenting into smaller clusters.

### Tuning Magnetic Properties through Post-annealing

We demonstrated through the previous pressure series that the magnetic properties in the Mn nitride system can be tuned continuously during the growth process. Next, we explore how magnetic properties can be controlled through post-annealing. Note that this second series of samples were grown at the same time and have the same layer structure as the *P*_N_ = 6% sample in the pressure series, except that they are capped with a 50 nm Ta layer instead of 5 nm Ti. Through its affinity for nitrogen, the thicker Ta layer acts as a nitrogen “getter”, which draws and stores nitrogen from the Mn nitride layers. Schematics in Figure 3a show the sample layer structure and the expected direction of nitrogen motion when annealed. As shown in Figure 3b, Mn_4_N, Mn_2_N, and Ta peaks can be seen in the reference (Ref) sample, which has not been through thermal treatment after growth. Interestingly, when compared with the *P*_N_ = 6% sample shown in Figure 1d, the Mn_3_N_2_ peak is missing in the Ref sample. This is likely caused by Ta’s strong affinity for nitrogen.^25,70,71^ Even though Ta is deposited at room temperature, it still spontaneously reacts with nitrogen and disrupts the Mn_3_N_2_ structure, similar to the redox reactions that occur in oxide systems with Gd.^12–14^

**Figure 3:**
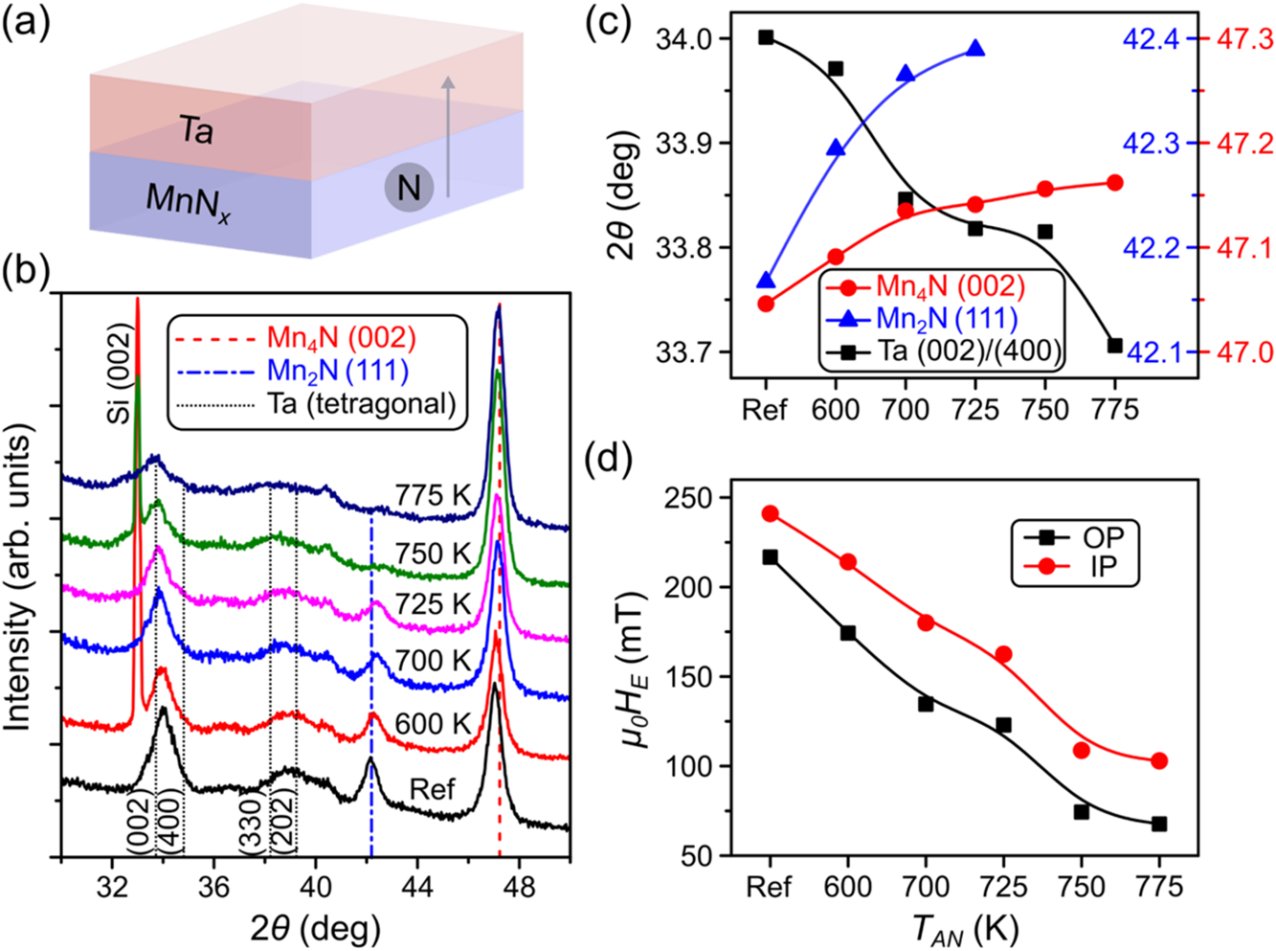
Structural and exchange bias variation for the annealing series. (a) Schematics of the layer structure MnN_*x*_ (60 nm)/Ta (50 nm), where MnN_*x*_ is composed of 20 nm Mn_3_N_2_ and 40 nm Mn fabricated with *P*_N_ = 6%. The arrow indicates the direction of nitrogen ion motion. (b) XRD 2θ-ω scans showing the phase evolution as *T*_AN_ changes. (c) Trends showing Mn_4_N (002) (red), Mn_2_N (111) (blue), and Ta (002)/(004) (black) peak position (extracted from (b)) variation as *T*_AN_ changes. (d) Exchange field dependence on *T*_AN_, extracted from the hysteresis loops measured at 5 K after +2 T field cooling from 300 K. Solid lines are guides to the eye. Error bars in (c, d) are smaller than the graph point size.

Individual samples cleaved from the same film as the Ref sample were then annealed in vacuum for 1 min at different annealing temperatures (*T*_AN_), referred to here as the “annealing series”. As *T*_AN_ increases, the Mn_2_N peak has the most notable change as it shifts to higher angles and eventually disappears at *T*_AN_ = 775 K (Figure 3b). Interestingly, the Ta peaks seem to simultaneously shift to lower angles and become broader with increasing temperature. The evolution of the different phases is evident in the plot of their peak positions shown in Figure 3c. Mn_2_N and Mn_4_N peaks both shift to higher angles as their lattice contracts after losing nitrogen to Ta. This process transforms the starting Mn_4_N/Mn_2_N layers back to the Mn_4_N single phase, opposite to the effect of increasing *P*_N_ shown in Figure 1d. Moreover, the Ta peaks shift to lower angles after absorbing nitrogen from the nitride phases which causes the Ta lattice to expand.^25^ These interpretations are consistent with the full range 2θ*-ω* and grazing incidence scans in Figure S4.

To study the EB, samples from the annealing series are field cooled from 300 to 5 K with a positive 2 T magnetic field. As *T*_AN_ increases, both OP and IP μ_0_ *H*_E_ decrease monotonically from 217 to 68 mT and 241 to 103 mT, respectively (Figure 3d). The decrease of EB is consistent with the reduction of the AF phase, Mn_2_N, as nitrogen moves into the Ta layer with annealing, and with the corresponding increase in the FiM Mn_4_N phase. These results demonstrate that the EB in all-Mn nitride systems can be controlled by driving nitrogen into a neighboring Ta layer with post-annealing. We also studied the room-temperature magnetization reversal behaviors of the annealing series using FORC, which shows the softer magnetic phase vanishing as nitrogen is removed from the Mn nitrides through annealing, consistent with the formation of more Mn_4_N phase. Results and detailed explanations can be found in Figure S5.

### Voltage Tuning of Magnetic Properties

Building on the annealing series results, voltage bias was used as another handle to drive ionic motion in the Mn nitride layers and to further understand how magnetic changes are affected by ionic motion. Shown in Figure 4a, MnN_*x*_ (15 nm)/Ta (10 nm) are deposited onto the Si substrate with thermally oxidized SiO_2_. The MnN_*x*_ layers are composed of a 5 nm Mn_3_N_2_ seed layer and 10 nm Mn deposited with *P*_N_ = 6%, keeping similar ratios between layers as the corresponding nitrogen and annealing series samples. The top electrical contact is made to the Ta layer, while the bottom contact is made to the *p*-type Si substrate. With this geometry, an electric field pointing from the top Ta to the bottom Si is established with positive voltage as shown in Figure 4a.^25,72^ A noticeable difference is observed in the IP hysteresis loops of the sample in the as-grown (AG) state and the voltage-conditioned (VC) state that was gated with +30 V for 1 h at room temperature (Figure 4b). Note that the hysteresis loops are measured on the same sample at 5 K right after positive 2 T field cooling before and after voltage conditioning. Closer examinations reveal an increase in *M*_S_ by 23% and a decrease in the coercivity (9%) and EB (2%). In the meantime, an intriguing kink near remanence appears in the VC state hysteresis. The increase in *M*_S_ indicates that more magnetic (ferrimagnetic or ferromagnetic) material has formed during voltage conditioning. The annealing series demonstrated that additional ferrimagnetic Mn_4_N can be produced by pulling nitrogen out of the Mn nitrides (Figure 3), and here, positive voltage conditioning is expected to drive nitrogen out of the Mn nitride and into the Ta layer, suggesting more FiM Mn_4_N has formed during voltage conditioning.

**Figure 4:**
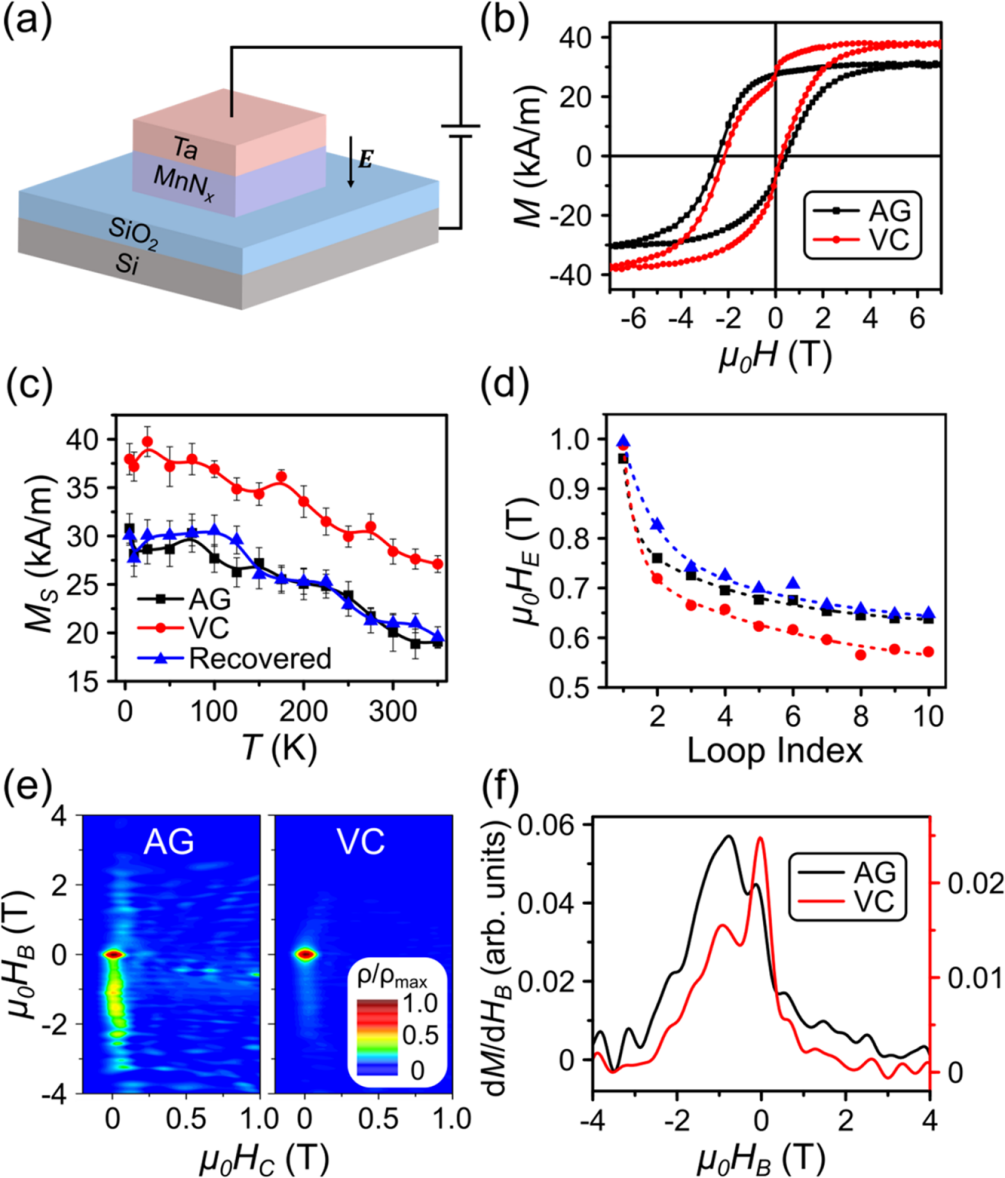
Voltage control of magnetism in the Mn nitrides. (a) Schematics showing the sample layer structure and the electric field direction with positive voltage bias. (b) Magnetic hysteresis loops of the sample before (AG, black curve) and after +30 V voltage conditioning (VC, red curve). (c) Temperature dependence of the saturation magnetization for the AG, VC, and recovered (blue curve, −45 V voltage conditioned) states. (d) Training effect for the AG, VC, and recovered states. Error bars are smaller than the graph point size: (e) FORC distributions and (f) bias field distributions for the AG and VC states. All loops are measured with an in-plane magnetic field at 5 K after field cooling the sample in a 2 T magnetic field from 300 K (b) and 380 K (c–e). Solid lines are guides to the eye, and dashed lines are the fits to eq 1.

We further studied the temperature dependence of *M*_S_, shown in Figure 4c. *M*_S_ is consistently larger in the VC state by ≈10 kA m^–1^ compared to the AG state of the sample from 5 to 350 K. This change is well above the measurement error indicated by the error bars and again suggests that the increase in magnetization is from the increased amount of Mn_4_N with a high Curie temperature around 745 K. Samples in the VC state were then gated with −45 V for 3 h to drive nitrogen back into the nitrides (recovered state). Interestingly, the blue curve in Figure 4c indicates that the *M*_S_ of the recovered state was reduced to similar values as the AG sample, suggesting the voltage-induced change in *M*_S_ is reversible. The temperature dependence of coercivity can be found in Figure S6.

To further confirm the voltage-induced change in the EB, we measured the training effect on the AG, VC, and recovered states, where ten consecutive loops were performed at 5 K after field cooling from 380 K. As shown in Figure 4d, there is little difference in the exchange field for the first loops. Interestingly, the gap between AG and VC exchange fields becomes wider after each loop, indicating the relaxation rate of the interfacial spins responsible for EB changes after voltage application. After fitting with eq 1, the equilibrium exchange fields (*H*_E_^∞^) are 622 mT and 525 mT for the AG and VC states, respectively. This is a significant decrease in the EB of 0.1 T (16%) at 5 K after voltage application. For the recovered state, *H*_E_^∞^ increases back to 613 mT, similar to the value of the AG state. These results show that the EB can be manipulated by voltage reversibly. Note that the large EB compared to the pressure series is due to the reduced film thickness since it is usually inversely proportional to the ferrimagnetic layer thickness.^51^

Furthermore, we also measured FORC with in-plane fields at 5 K after field cooling and field training to highlight the voltage-induced changes in magnetic properties (Figure 4e). The FORC distribution for the AG state displays a prominent vertical ridge feature, located along the coercivity (μ_0_ *H*_C_) = 0 axis. Close examination reveals a sharp peak feature centered on the origin. This feature is typically associated with magnetically soft particles which reverses via single domain rotation.^73^ This suggests that some parts of the film are not exchange-biased, likely due to the small size of the magnetic clusters. Besides the peak, there is also a large spread along the bias (μ_0_ *H*_B_) axis. This vertical spread is mainly located on the negative μ_0_ *H*_B_ axis, which is characteristic of EB systems. This is consistent with the major hysteresis loops that shows the sample is exchange-biased in the negative field direction. Interestingly, the VC state FORC distribution is drastically different from that of the AG state (Figure 4e). All the features are still along the μ_0_ *H*_C_ = 0 axis, but the spread has been reduced. Additionally, the feature near origin now dominates the signal. These features suggest that the EB has been reduced by voltage application, which is also consistent with the results shown in Figure 4d. Moreover, the growth of the FORC feature near the origin can also be associated with the kink near remanence in the hysteresis loop for the VC state (Figure 4b). This is because the Mn_4_N formed through voltage-induced nitrogen motion likely exists as small clusters, which would contribute to the softer phase that switches near remanence. Similar voltage-induced changes were also observed in another Co_*x*_Mn_1–*x*_N system.^72^ Moreover, these changes can be highlighted by the bias field distribution plots in Figure 4f, which are done by projecting the FORC distribution onto the μ_0_ *H*_B_ axis and integrating along the μ_0_ *H*_C_ axis. Both plots are asymmetric around μ_0_ *H*_B_ = 0, indicating the samples are both exchange-biased. Nevertheless, the AG state has a much more pronounced peak around μ_0_ *H*_B_ = −1 T than the VC state, indicating larger EB. On the other hand, the peak around μ_0_ *H*_B_ = 0 in the VC state gets larger after gating, indicating the increase of magnetic regions that are not exchange-biased. These changes are also consistent with our interpretation that EB is reduced through voltage-induced nitrogen ionic motion, which causes the formation of more ferrimagnetic Mn_4_N, likely from the AF Mn_2_N.

### Confirming Nitrogen Motion Using Polarized Neutron Reflectivity (PNR)

To gain a deeper understanding of the nitrogen ionic motion within the thin film heterostructures, we have conducted PNR experiments on samples from the pressure, annealing, and gating series (Figure 5). The sensitivity of PNR benefits from the scattering length density (SLD) contrast that is produced by small variations in nitrogen concentrations in MnN_*x*_,^26^ as the increase in nitrogen concentration from pure Mn to Mn_3_N_2_ produces nuclear SLD (ρ) variation from −2.98 × 10^–6^ Å^–2^[1 Å = 10^–10^ m] to 1.38 × 10^–6^ Å^–2^, respectively. This allows for more accurate identification of the expected MnN_*x*_ phases as a function of depth, normal to the substrate, and the nitrogen ionic motion within the heterostructures. Expected ρ values for other potential MnN_*x*_ phases in the system, Ta, TaN, and Ta_2_O_5_ can be found in Table S3. Fits of the PNR data for each sample using the chosen model are shown in Figures S7–S13, along with other excluded fitting models and a discussion of how the best model was chosen.

**Figure 5:**
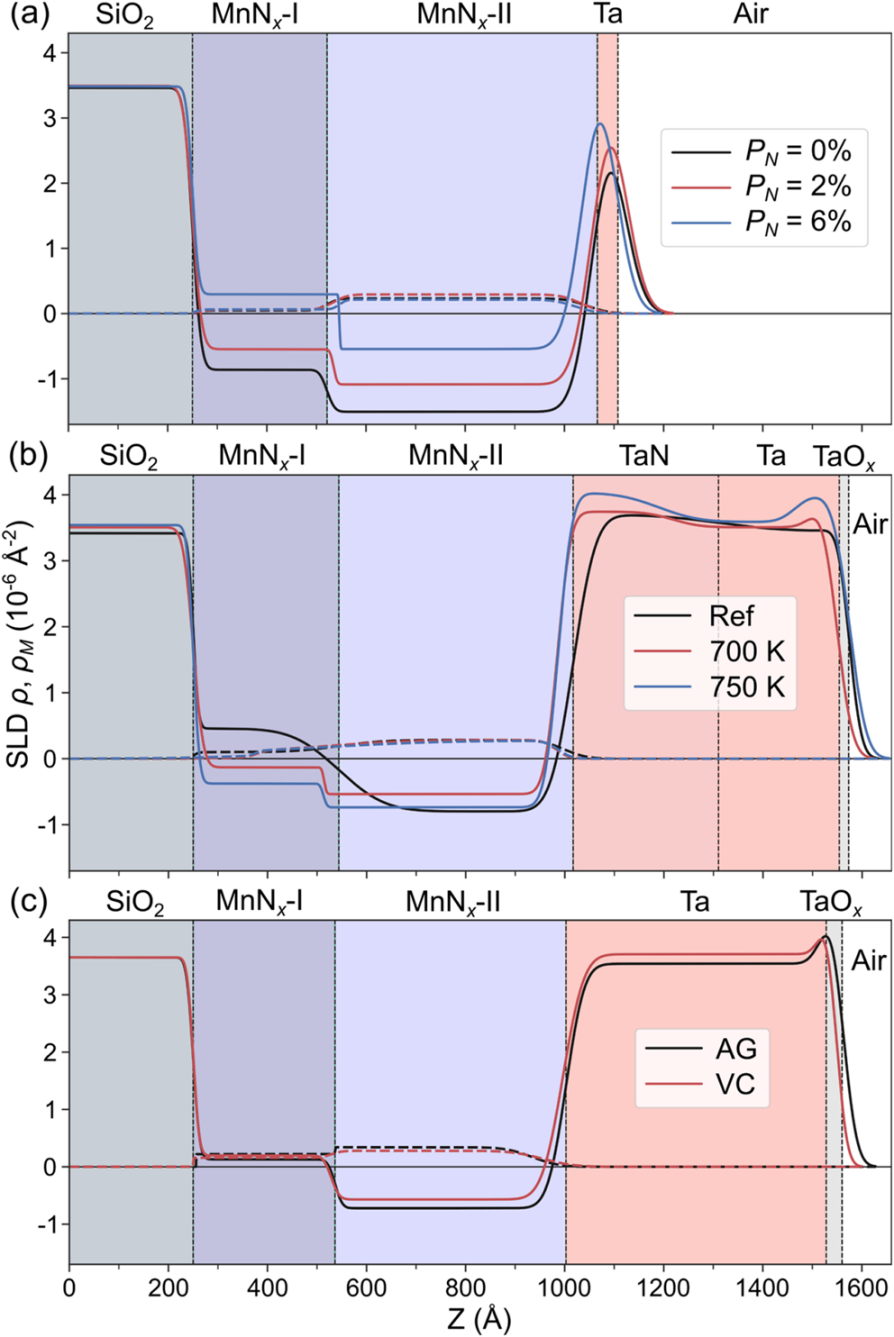
Scattering length density (SLD) depth profile from the polarized neutron reflectivity (PNR) studies. SLD depth profiles of (a) *P*_N_ = 0% (black), 2% (red), and 6% (blue) samples from the pressure series, (b) as-grown (Ref) (black), 700 K (red), and 750 K (blue) annealed samples from the annealing series, and (c) AG (black curve) and +30 V gated (red curve) samples from the gating series. Solid and dashed lines represent the nuclear and magnetic SLD, respectively. The background colors indicate locations of various layers in the sample heterostructure. All measurements were done in a 2.75 T IP magnetic field at room temperature.

For these room-temperature PNR measurements in an IP 2.75 T field, we considered three representative samples from the pressure series (*P*_N_ = 0, 2, and 6%, capped with Ta instead of Ti), three representative samples from the annealing series (AG, 700 K annealed, and 750 K annealed), and two representative samples from the gating series (AG and voltage conditioned). Note that the samples used for neutron experiments are thicker and were grown separately from the samples used for magnetometry and XRD studies. The Mn nitride layers in all eight samples can be fitted with a two-layer model for the depth dependence of the SLD, where each layer’s thickness is comparable to the nominal thickness of the Mn_3_N_2_ seed layer (27 nm) and Mn layer (51 nm) deposited with various nitrogen partial pressures. Fitting values for the chosen model discussed here can be found in Tables S4–S6.

For the pressure series samples (Figure 5a), the bottom MnN_*x*_-I layer in the *P*_N_ = 0% sample is modeled with a significantly lower value of ρ than would be expected for the as-grown Mn_3_N_2_. Additionally, the top MnN_*x*_-II layer, despite being deposited in a pure Ar environment, was modeled with a much higher ρ compared to that of the as-grown Mn. These differences from the expected ρ in both MnN_*x*_ layers are consistent with the as-deposited Mn (MnN_*x*_-II layer) acquiring nitrogen from the bottom Mn_3_N_2_ (MnN_*x*_-I layer) during growth to form Mn_4_N and nitrogen-deficient Mn_4_N, respectively. As *P*_N_ is increased to 2 and 6%, the bottom MnN_*x*_-I layer’s ρ increases significantly, which may represent Mn_4_N mixed with an increasing amount of Mn_2_N. Similarly, ρ in the top MnN_*x*_-II layer also significantly increases with increasing *P*_N_ in the 2 and 6% samples, representative of Mn_4_N and mixed phases of Mn_4_N and Mn_2_N, respectively. This is consistent with the trends seen in the XRD and magnetometry results. The modeled ρ for the Ta layer also increases with *P*_N_, varying from that expected for Ta, and is likely caused by more nitrogen moving into the Ta layer as *P*_N_ increases, along with oxidation. These changes in ρ are all statistically significant as the 95% confidence intervals (CI) of the modeled ρ have no overlap (see Tables S4–S6). Unlike ρ, the change in the magnetic SLD (ρ_M_) is less conclusive between the samples, which could be attributed to the small *M*_S_ (<85 emu/cc) and small variation (<25%) in *M*_S_ when *P*_N_ is varied. Nevertheless, a consistently larger ρ_M_ is seen in the top MnN_*x*_-II layer compared to that of the bottom MnN_*x*_-I layer within each of the pressure series samples. This suggests that most of the magnetic signal is coming from the MnN_*x*_-II layer that was deposited onto the Mn_3_N_2_ seed layer.

The PNR results for the annealing series are summarized in Figure 5b. Note that the annealing series samples were all grown with the same parameters as the pressure series sample, *P*_N_ = 6%, with the only difference being a thicker Ta layer (50 nm) on top for nitrogen storage. The samples were grown on a single wafer and cut into individual samples prior to annealing, and the reference sample (Ref) was left unannealed. In the Ref sample, the MnN_*x*_ layers have ρ values that are comparable with the 6% sample in the pressure series, indicating consistency between sample growth. After the annealing at 700 K for 1 min, the ρ drops considerably in the MnN_*x*_-I layer. When the sample is annealed at 750 K, ρ decreases further in the MnN_*x*_-I layer. A smaller increase followed by a decrease in ρ is noted in the MnN_*x*_-II layer, but these changes did not reach statistical significance. The Ta layer in all three conditions (Ref, 700 and 750 K) is modeled with three sublayers that are referred to as TaN_*x*_, Ta, and TaO_*x*_ in Figure 5b. From the Ref sample, the TaN_*x*_ layer ρ increases with an increasing annealing temperature. Considering this together with the decreasing ρ in MnN_*x*_-I, the models are consistent with more nitrogen moving out of MnN_*x*_ and into the Ta layer with an increasing temperature. A lack of a significant change in the MnN_*x*_-II layer may be caused by the competing diffusion of N in the layer as it draws N from the MnN_*x*_-I and loses N to the Ta layer, which may lead to a smaller change in overall N content. TaO_*x*_ is indicated in each condition with an increase in ρ at the surface of the sample, likely caused by the presence of oxygen in the annealing chamber or from exposure to air. It should be noted that the apparent decrease in MnN_*x*_ total thickness and increase in total Ta thickness is consistent with nitrogen moving out of MnN_*x*_ and into Ta, although the change is not statistically significant in the models.

In the gating series, PNR measurements were performed on one control sample (AG) and another gated with +30 V, grown with the same parameters as the Ref sample from the annealing series, and cut from the same wafer after growth. The best model that fits the two samples are very similar, as shown in Figure 5c, which suggests the nitrogen motion that is induced by voltage is less significant compared to that in the pressure and annealing series. Moreover, the magneto-ionic effect is greatly affected by sample thickness, where the effect can be much weaker in thicker samples. Nevertheless, statistically significant changes can be identified by careful inspection of the ρ variation. The bottom MnN_*x*_-I ρ for both gated series samples is similar, and statistically significant differences between them cannot be determined as their 95% CI largely overlap. On the other hand, the top MnN_*x*_-II variation reaches statistical significance, with an increase in ρ after gating, suggesting nitrogen has moved into this layer from the MnN_*x*_-I. The Ta layer also has a statistically significant increase in ρ after voltage conditioning. Considering these factors, we can speculate that nitrogen has moved into the top MnN_*x*_-II and Ta layer from the bottom nitrogen-rich MnN_*x*_-I layer after applying voltage, as it is the only available source of nitrogen and is consistent with the expected nitrogen ion motion with positive voltage gating.

## Conclusions

In this study, we have demonstrated an all-Mn-nitride magneto-ionic system whose magnetic phases can be dynamically tuned to be AF only, AF/FiM, or FiM only by addition or removal of nitrogen. By controlling the nitrogen partial pressure during deposition and thermally induced nitrogen motion facilitated by an adjacent tantalum layer, the magnetic characteristics of the nitride system can be modulated substantially. Specifically, the resultant exchange bias effect can be increased by over an order of magnitude from 23 to 234 mT as the nitrogen partial pressure is increased during deposition. Subsequently, exchange bias can be reduced by over 70% through post-annealing, where nitrogen moves out of the Mn nitride and into the neighboring Ta layer. XRD, TEM, and magnetometry studies confirmed the phase transformations from Mn_4_N phase to mixed phases of Mn_4_N/Mn_2_N and Mn_4_N/Mn_2_N/Mn_3_N_2_ with nitrogen addition, and the reverse transformation with nitrogen removal. Furthermore, we demonstrate the voltage control of magnetic properties in this Mn nitride system through room-temperature solid-state gating. Specifically, an increase in saturation magnetization by 23% and a decrease in exchange bias by 0.1 T (16%) at 5 K were observed when nitrogen ions were driven out of the Mn nitrides and into a neighboring Ta layer using a positive voltage application. This is attributed to the formation of more Mn_4_N, likely transformed from Mn_2_N. Subsequently, the changes can be reversed upon negative voltage conditioning. Additionally, polarized neutron reflectometry confirmed the expected nitrogen motions during deposition, post-annealing, and voltage application.

These results demonstrate an all-Mn-nitride system in which the magnetic phases and their properties, such as large exchange bias, can be dynamically and magneto-ionically tuned through deposition conditions, post-annealing, and solid-state voltage application. They illustrate a promising platform for energy-efficient and sustainable spintronic applications. They also open up potential avenues for achieving magnetic immunity in magnetic data storage and memory. For instance, information can be written in the FiM state and securely stored in the AF state, which exhibits no stray field, thereby ensuring immunity to external magnetic interference. Furthermore, we propose a few ways to accelerate magneto-ionic actuation. First, dielectric scaling, which involves the reduction of the dielectric layer thickness, can increase the effective electric field, thereby enhancing the ionic mobility and significantly shortening the magneto-ionic switching time. Second, resistivity tailoring through controlled doping of the Mn nitrides can modulate the film’s electrical resistivity, effectively redistributing the potential drop to the active layer and facilitating faster ionic migration.^34,74^ Third, defect engineering through deliberate introduction of point and extended defects, such as nitrogen vacancies and grain boundaries, provides low-energy diffusion pathways, simultaneously reducing the ion migration distance and lowering the activation barrier for ionic transport.^75^ When these strategies are employed synergistically, they may offer a robust pathway toward achieving rapid and energy-efficient magneto-ionic switching without compromising device performance or stability.

## Methods

### Fabrication

Mn_3_N_2_ seed layers of varying thickness (*y* nm) were DC reactively sputtered onto a Si substrate with a 285 nm SiO_2_ layer at 723 K substrate temperature in an ultrahigh-vacuum chamber (<7 × 10^–6^ Pa base pressure) with an Ar:N_2_ ratio of 1:1 and 0.67 Pa sputtering pressure. The Mn_3_N_2_ seed layer was vacuum annealed for 30 min at 723 K to enhance its crystallinity. Subsequently, nominally 2 × *y* nm Mn was deposited onto the Mn_3_N_2_ layer at the same 723 K substrate temperature with nitrogen partial pressures (*P*_N_) varying from 0 to 6%, where $P_{N}=\frac{N_{2}\;flow\;rate}{Ar+N_{2}\;flow\;rate}\times 100\%$ . After deposition, substrate heating was turned off immediately, and the samples were cooled to room temperature before depositing a Ti or Ta capping layer. All samples were fabricated using this method, and only thickness *y*, *P*_N_, and capping layer vary between the sample series.

#### Pressure Series

*y* = 20 for the samples used for magnetometry and XRD, and the capping layer is 5 nm Ti. *P*_N_ varies from 0 to 6%. *y* = 27 for the samples used for the PNR experiments.

#### Annealing Series

*y* = 20 for all of the samples used for magnetometry and XRD. *y* = 27 for the samples used for PNR experiments. *P*_N_ is fixed at 6% for all samples. Samples were capped with 50 nm Ta and annealed at different temperatures for 1 min at 27 Pa inside a Quantum Design superconducting quantum interference device (SQUID) MPMS3 sample chamber in a helium environment.

#### Gating Samples

*y* = 5, and a 10 nm Ta capping layer is used for all the samples studied by magnetometry. *y* = 27, and a 50 nm Ta capping layer is used for the PNR samples. *P*_N_ is fixed at 6% for all samples. The films were wet-etched into 5 mm × 5 mm pieces after deposition. Voltage bias is applied at room temperature under atmosphere condition.

### Structural Characterizations

XRD was performed on a Panalytical X’Pert^3^ MRD system with both symmetric 2θ-ω and grazing incidence scans with a 0.5° incidence angle. X-ray source is Cu K_α_ with a 0.154 nm wavelength. The cross-sectional TEM lamella was fabricated using the Helios G4 UX FIB system (Thermo Fisher Scientific), equipped with a Ga^+^ beam source. The thickness of the lamellae was less than 100 nm. The high-resolution HAADF-STEM images and EDS results were obtained using an FEI Titan Themes Cubed G2 300 TEM equipped with a probe corrector under 300 kV accelerating voltage.

### Magnetic Characterizations

Hysteresis measurements were performed on a Quantum Design SQUID MPMS3 magnetometer. Exchange bias was measured at 5 K by first field cooling the sample from 300 and 380 K in a positive 2 T magnetic field unless otherwise stated. Exchange fields (*H*_E_) and coercivities (*H*_C_) are calculated by *H*_E_ = |(*H*_L_ + *H*_R_)/2|, and *H*_C_ = (*H*_R_ – *H*_L_)/2, where *H*_L_ and *H*_R_ are the hysteresis loop’s lower and upper fields at which the magnetization is zero.

Room-temperature first-order reversal curve (FORC) measurements with out-of-plane fields were carried out in a Princeton Measurement Corporation MicroMag 3900 vibrating sample magnetometer. Low-temperature FORC studies with in-plane fields were carried out in an MPMS3 magnetometer. Samples were first saturated in a positive magnetic field. Subsequently, measurements were taken at each field step from a reversal field (*H*_r_) back to saturation in uniform steps. This process was then repeated at different *H*_r_ values to fill the interior of the hysteresis loop, creating a family of FORCs. The FORC distribution is defined using the following equation:

$$\rho \left(H,H_{r}\right)\equiv -\frac{1}{2M_{S}}\frac{\partial^{2}M\left(H,H_{r}\right)}{\partial H\partial H_{r}}$$

where *M*_S_ is the saturation magnetization, and *M*(*H*, *H*_r_) is the magnetization at the applied field *H* with reversal field *H*_r_. The FORC distribution can also be represented in terms of local coercive field and bias field (*H*_C_, *H*_B_) defined by *H*_C_ = (*H* - *H*_r_)/2 and *H*_B_ = (*H* + *H*_r_)/2, respectively.

### Polarized Neutron Reflectivity (PNR)

PNR measurements were carried out on the POLREF instrument at the ISIS Rutherford Appleton Laboratory in Didcot, England. The data for the experiment can be found at 10.5286/ISIS.E.RB2320315-1.^76^ Room-temperature measurements were taken for each sample with an applied in-plane magnetic field of 2.75 T. The incident neutron beam was polarized + or −, corresponding to neutron spins parallel or antiparallel to the magnetic field. For this experiment, only non-spin-flip specular reflectivities (*R* ^+^ and *R* ^–^) were measured with respect to the wave vector transfer, *Q*. The data were reduced using Mantid data reduction software,^77,78^ and Refl1D software packages were used to fit the data.^79,80^ Error bars for the fitted parameters were determined with a Markov chain Monte Carlo method by using the BUMPS software package.

## Supplementary Material



## Notes

XRD results; temperature and cooling dependence of exchange bias; additional FORC results; and fitting parameters and other fitting models used for PNR (PDF)
