# Supplementary material for: Reconfigurable All-Nitride Magneto-Ionics
Source: ACS Nano. 2025 May 7;19(21):20072–83. doi: 10.1021/acsnano.5c04013 (PMC12139039; doi:10.1021/acsnano.5c04013)
Supplement: Supplementary file 1 [file nn5c04013_si_001.pdf]

Supporting Information for
Reconfigurable All-Nitride Magneto-Ionics

*Zhijie Chen,[1,] * Christopher J. Jensen,[1, 2] Chen Liu,[3] Yijing Liu,[1] Christy J. Kinane,[4] Andrew John Caruana,[4] Alexander J. Grutter,[2] Julie A. Borchers,[2] Xixiang Zhang,[3] and Kai Liu[1,] **

[1]Physics Department, Georgetown University, Washington, DC 20057, USA
[2]NIST Center for Neutron Research, National Institute of Standards and Technology, Gaithersburg, MD 20899, USA
[3]King Abdullah University of Science & Technology, Thuwal 23955-6900, Saudi Arabia
[4]ISIS Neutron Facility, STFC Rutherford Appleton Laboratory, Chilton, OX11 0QX, Oxfordshire, UK

* E-mail: zc150@georgetown.edu, kai.liu@georgetown.edu

This Supporting Information document includes the following sections:

1. Pressure series X-ray diffraction
2. Temperature and cooling field dependence of exchange bias for the pressure series
3. Pressure series first order reversal curve (FORC)
4. Fitting parameters
5. Annealing series X-ray diffraction
6. Annealing series FORC
7. Temperature dependence of coercivities for the gating series
8. Polarized Neutron Reflectometry

1. Pressure series X-ray diffraction

Further X-ray measurements including full range 2θ-ω and grazing incidence scans were done on the pressure series samples with nitrogen partial pressure (P_N) varying from 0% to 6% during Mn deposition on top of 20 nm Mn_3N_2 seed layers. Full range 2θ-ω scans are done with a 1° ω offset to suppress the substrate peak. As shown in Figure S1a, these scans show the same information as Figure 1d in the main text, where the peaks for different phases are mainly located between 40° - 50°. Moreover, a scan on the Mn_3N_2 seed layer is also included (top curve), which shows the Mn_3N_2 is (020) oriented, consistent with previous reports.[1, 2]

Grazing incidence scans were also done on the same pressure series, with the incidence X-ray at a fixed angle ($\omega = 1°$) while only 2θ is changing. These asymmetric scans allow us to detect other crystal planes tilted away from the sample surface. Starting with P_N = 0%, all the peaks in the scan belong to the Mn_4N phase and all the peaks from Mn_4N are accounted for (PDF: 00-001-1202). This suggests that P_N = 0% sample only contains the Mn_4N phase. As P_N increases to 2%, two peaks, (111) and (300), from the $Mn_2N_{0.86}$ phase show up (PDF: 01-071-0200), indicating that more nitrogen is incorporated into the nitrides and the Mn_2N phase forms. As P_N increases to 5%, (020) and (220) peaks from the Mn_3N_2 phase show up, indicating some Mn_3N_2 is recovered when there is enough nitrogen during deposition. Additionally, a reference pattern for the Mn_3N_2 seed layer is also presented (Figure S1b top curve). All peaks in the scan belong to the Mn_3N_2 phase, confirming the seed layer is a Mn_3N_2 single phase.

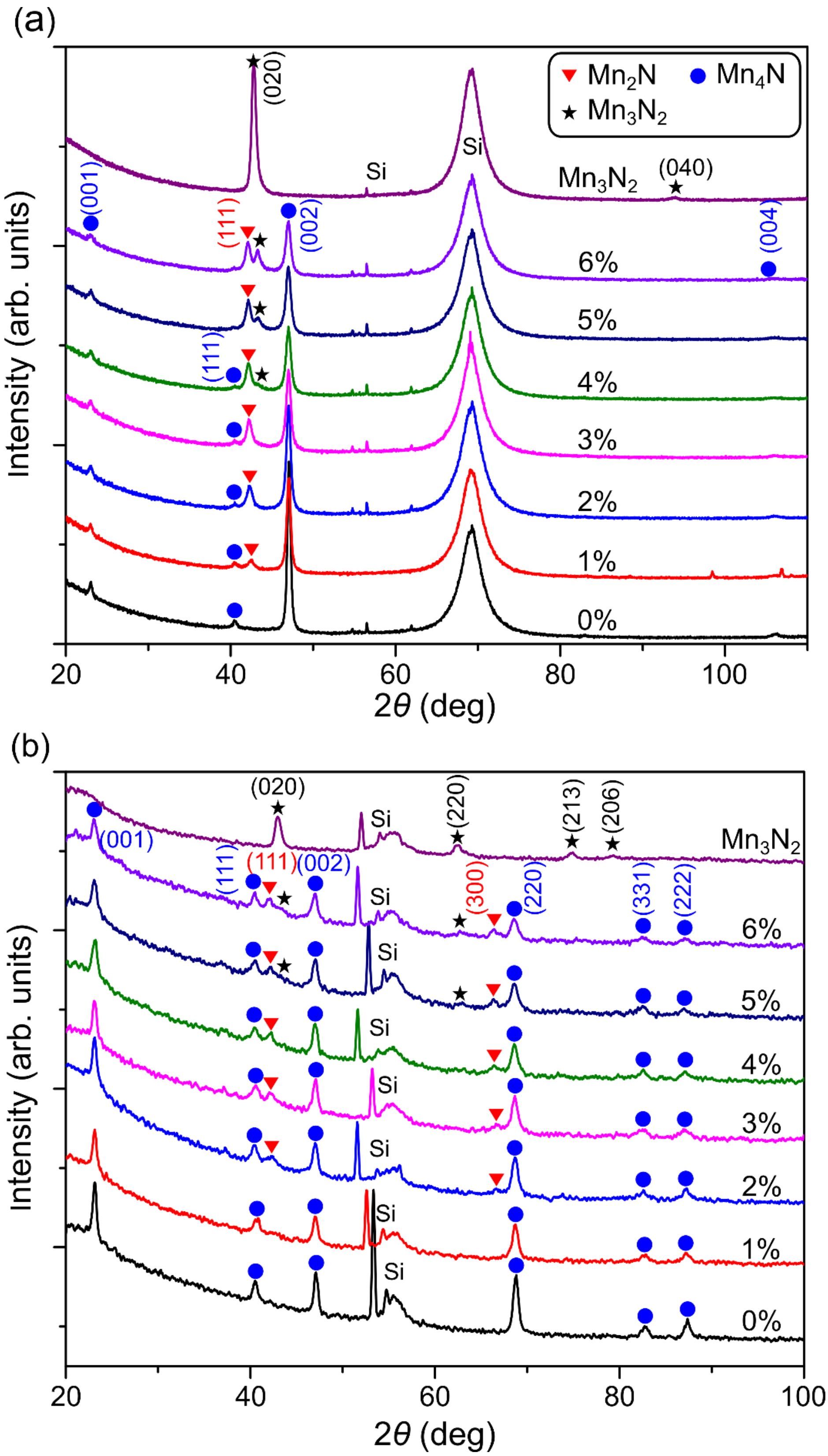

Figure S1. X-ray diffraction results on the pressure series. (a) Full range 2θ-ω and (b) grazing incidence X-ray scans for the pressure series with P_N from 0% to 6%, and a single Mn_3N_2 seed layer. P_N is the nitrogen partial pressure during Mn deposition on top of 20 nm Mn_3N_2 seed layers. X-ray source is Cu K-alpha with 0.154 nm wavelength.

2. Temperature and cooling field dependence of exchange bias for the pressure series.

To further elucidate the origin of the exchange bias effect, we have studied its temperature dependence. Samples were initially field cooled from 380 K to 5 K in a positive 2 T IP magnetic field and field trained with ten hysteresis loops. Afterward, a hysteresis loop was recorded at each temperature step as it warms back to 350K. Exchange field ($\mu_0 H_E$) was extracted and plotted as a function of temperature for samples with different P_N shown in Figure S2a. $\mu_0 H_E$ monotonically decreases in all samples and vanishes around 325 K. We can further fit the $\mu_0 H_E$ using the following exponential function,[3]

$$\mu_0 H_E(T) = \mu_0 H_E^0 * \exp\left(-\frac{T}{\tau}\right), \quad (1)$$

where $\mu_0 H_E^0$ is the extrapolation of $\mu_0 H_E$ to absolute zero temperature and τ is a constant. The fitted curves are the dotted lines in Figure S2a, and the fitting parameters can be found in Table S2. This exponential temperature-dependent decay of H_E has been seen in systems with frustrated spins caused by competing magnetic interactions.[3-6] Moreover, in the temperature-dependent coercivity ($\mu_0 H_C$) curve (Figure S2b), a small peak at low temperature can be seen across the samples. This peak in $\mu_0 H_C$ is normally associated with rotatable AF spins or glassy spins.[6, 7] Below the temperature where the peak appears, the AF spins are completely frozen and lead to reduced coercivity. Upon close examination, the peak in $\mu_0 H_C$ also shows up at higher temperatures with increasing P_N, from 18 K for P_N = 0%, 21 K for 1%, 22 K for 2%, to 26 K for 6% sample. This trend indicates that the Mn nitride systems have more glassy spins as the nitrogen concentration increases.

This interpretation is further corroborated by examining the exchange bias with different cooling fields. Samples were first demagnetized at room temperature and then cooled down to 5 K in a positive IP magnetic field (cooling field). As shown in Figure S2c, $\mu_0 H_E$ rises rapidly and peaks before decreasing slowly as the cooling field increases. This behavior in $\mu_0 H_E$ is again often associated with exchange bias systems containing glassy spins.[8-11] The initial increase in $\mu_0 H_E$ as the cooling fields increase is due to an increased FiM alignment. However, as the cooling field gets larger, Zeeman energy is significant enough to compete with the frustrated exchange interaction which leads to glassy spins. As the glassy spin in the system gets reduced due to better alignment with the large magnetic fields, their contribution to the exchange bias gets reduced. The fields at which $\mu_0 H_E$ peaks also increase monotonically as P_N increases from 0.6 T for P_N = 0%, 0.8 T for 1%, 1 T for 2%, to 2.2 T for 6%, indicating the higher P_N sample contains more glassy spins which requires larger field to align. This is

consistent with the previous interpretation about the $\mu_0 H_C$ maxima (Figure S2b). Moreover, the cooling fields dependence of $\mu_0 H_C$ shows a similar trend as the $\mu_0 H_E$, where the peak field gets pushed to high fields for samples with higher nitrogen concentrations (Figure S2d).

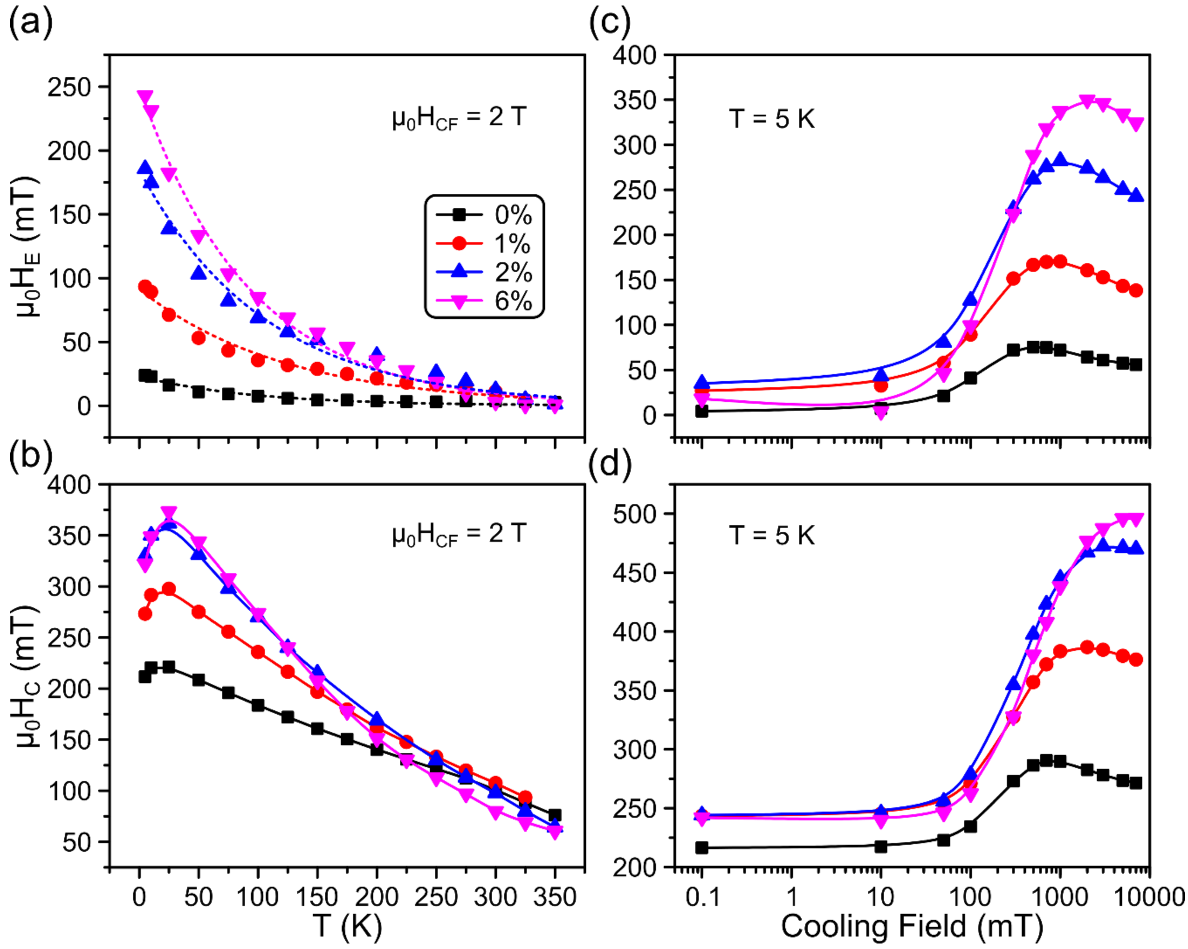

Figure S2. Temperature and cooling field dependences of exchange bias for the pressure series. The temperature (T) dependence of (a) exchange field ($\mu_0 H_E$) and (b) coercive field ($\mu_0 H_C$). They are extracted from the field-trained hysteresis loops measured as the temperature warms from 5 K to 350 K after field cooling to 5 K in +2 T in-plane magnetic field from 380 K. Dotted lines are fits to Eq. 1. The cooling field dependence of (c) $\mu_0 H_E$ and (d) $\mu_0 H_C$, extracted from the hysteresis loops measured at 5 K after cooling from 300 K with different in-plane fields. Error bars are smaller than the graph point size.

3. Pressure series first order reversal curve (FORC)

To study the room temperature magnetic properties of the pressure series samples. FORCs were taken on the P_N = 0%, 2%, 4%, and 6% samples with out-of-plane magnetic field at room temperature. As shown in Figure S3a, the 0% FORCs exhibit a square hysteresis with each FORC returning to positive saturation in a generally horizontal way. The corresponding FORC distribution shows one prominent feature at $\mu_0 H_C$ = 300 mT (Figure S3e), which

indicates a high anisotropy phase with perpendicular magnetic anisotropy.[1, 12] This is consistent with the interpretations from the X-ray results in Figure 1d and S1 that the 0% sample is a Mn_4N single phase. As P_N increases to 2%, 4%, and 6%, the FORC paths get more slanted (Figs. S3b, c, and d). Another vertical ridge in the FORC distribution centered around $\mu_0 H_C$ = 0 T, which corresponds to reversible switching,[12, 13] also emerges (Figure S3f, g, and h). In the meantime, the main FORC feature at the higher H_C starts to spread out and becomes less prominent. Interestingly, the spread of the vertical ridge along the $\mu_0 H_B$ axis gets lager as P_N increases, suggesting an increase of dipolar interactions within the film.[14, 15] This indicates that some of the Mn_4N are becoming magnetically softer and possibly broken up into smaller gains as other Mn nitride phases start to form (Mn_2N and Mn_3N_2), resulting from incorporation of more nitrogen into the system.

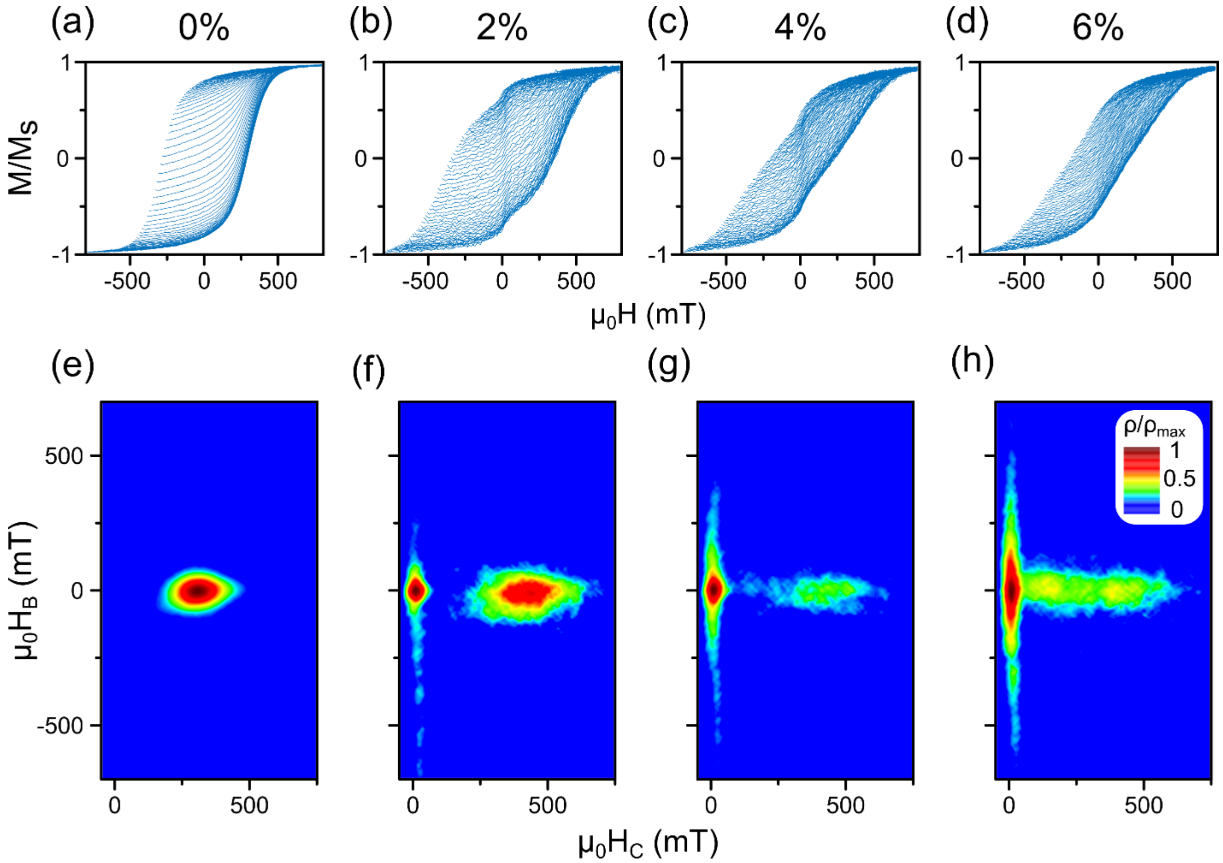

Figure S3. First-order reversal curve study on samples from the pressure series. Families of FORCs (top row) and FORC distributions (bottom row) for [(a) and (e)] P_N = 0%, [(b) and (f)] P_N = 2%, [(c) and (g)] P_N = 4%, [(d) and (h)] P_N = 6% samples in the pressure series. Magnetic field is along the out-of-plane direction.

4. Fitting parameters

The fitting parameters for the training effect shown in Figure 2d in the main text are included in Table S1. The fitting parameters for the temperature dependence of the exchange field shown in the supplementary information Figure S2a are included in Table S2. Errors were obtained from the fit.

Table S1. Fitting parameters for the pressure series training effect.

Sample	H_E^{∞} (mT)	A_F (mT)	A_R (mT)	P_F	P_R
0%	22.73 ± 0.35	262.42 ± 34.69	19.59 ± 2.37	0.46 ± 0.04	3.07 ± 0.41
1%	87.93 ± 1.41	1217.16 ± 2351.29	51.61 ± 10.40	0.29 ± 0.02	2.81 ± 0.06
2%	177.12 ± 1.55	519.34 ± 79.71	52.79 ± 6.87	0.49 ± 0.05	3.50 ± 0.63
6%	234.08 ± 1.66	782.62 ± 247.02	66.48 ± 10.87	0.42 ± 0.07	3.05 ± 0.56

Table S2. Fitting parameters for the temperature dependence of exchange field for the pressure series.

Sample	H_E^0 (mT)	τ
0%	23.1 ± 1.3	94.8 ± 9.9
1%	91.3 ± 3.1	122.3 ± 7.5
2%	185.0 ± 5.5	105.3 ± 6.2
6%	250.1 ± 5.1	92.3 ± 3.5

5. Annealing series X-ray diffraction

Further X-ray measurements including full range 2θ-ω and grazing incidence scans were done on the annealing series samples with annealing temperature varying from room temperature (reference) to 775 K. These samples are grown the same way as the P_N = 6% sample in the pressure series, but with a 50 nm Ta top layer acting as a nitrogen absorber. Full range 2θ-ω scans in Figure S4a are done with a 1° ω offset to suppress the substrate peak. These scans show the same information as Figure 4b in the main text, where the peaks for different phases are mainly located between 30° - 50°. Grazing incidence scans shown in Figure S4b were done with the incidence X-ray fixed at 1° ω angle, while only the 2θ angle is changing. The most prominent peaks in the grazing incidence scans are from Ta due to its larger density. Note the sample sizes in the annealing series are much smaller than in the pressure series because of experimental constraints. Thus, the Mn nitride peaks are smaller compared to the pressure series in Figure S1b. Nevertheless, the Ta peaks in Figure S4b decrease and shift to the lower angles as the annealing temperature increases, consistent with the interpretation for

main text Figure 3 that Ta absorbs the nitrogen after annealing. This causes Ta lattices to expand and turn into Ta nitrides.

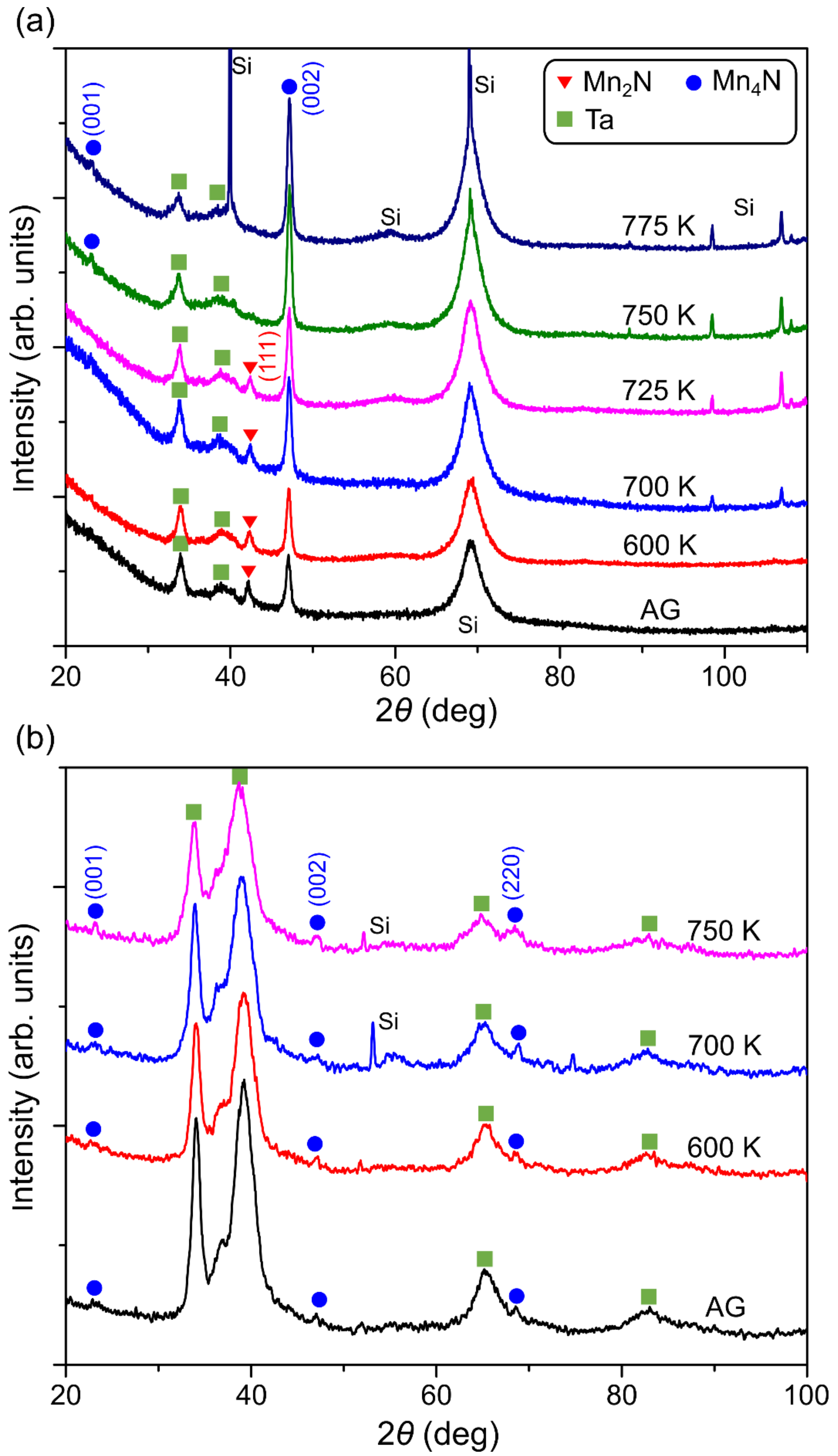

Figure S4. X-ray diffraction results on the annealing series. (a) Full range 2θ-ω and (b) grazing incidence X-ray scans for the annealing series with annealing temperatures from 600 K to 775 K labeled next each curve. AG is the as-grown reference sample without any post-annealing. X-ray source is Cu K-alpha with 0.154 nm wavelength.

6. Annealing series FORC

We also did FORC studies on the annealing series as shown in Figure S5. Interestingly, the FORC path and distributions exhibit the opposite trends as annealing temperature increases compared to increasing P_N. As shown in Figure S5a, the unannealed reference sample shows a slanted FORC, with the corresponding FORC distribution (Figure S5e) showing two main features, one vertical ridge at $\mu_0 H_C$ = 0 T and a horizontal feature at $\mu_0 H_C$ = 0.4 T. As the annealing temperature increases, the FORC paths become more horizontal (Figure S5 b, c, and d), while the vertical ridge at $\mu_0 H_C$ = 0 T is vanishing, and the horizontal feature shifts to higher $\mu_0 H_C$ of 0.55 T, 0.65 T, and 0.68 T (Figure S5 f, g, and h). These results indicate that the film becomes Mn_4N single phase as the nitrogen is removed from the system and the Mn_4N crystallinity improves (larger coercivity) when the annealing temperature increases. This process is opposite to increasing P_N during deposition which increases the nitrogen concentration in the system as shown in Figure S3.

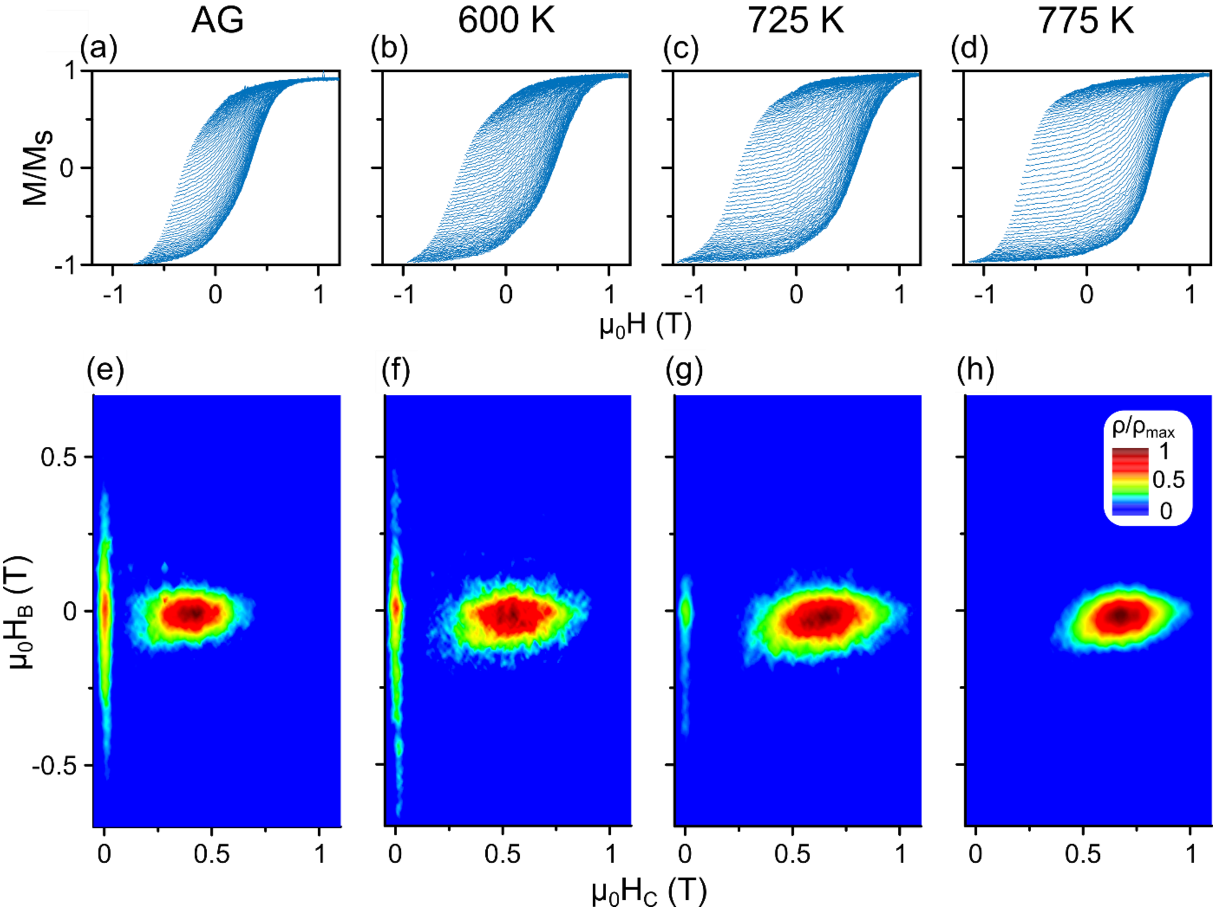

Figure S5: FORC study on samples from the annealing series. Families of FORCs (top row) and FORC distributions (bottom row) for [(a) and which] as-grown sample, [(b) and (f)] T_{AN} = 600 K, [(c) and (g)] T_{AN} = 725 K, [(d) and (h)] T_{AN} = 775 K samples in the annealing series. T_{AN} is the post-annealing temperature.

7. Temperature dependence of coercivities for the gating series

The temperature dependence of the coercivity is also plotted in Figure S6. The VC state coercivity is considerably smaller than the AG state of the sample at all temperatures, likely because there is less coercivity enhancement effect as the EB gets smaller. Interestingly, the coercivities first peak at some lower temperatures and then decrease as temperature increases, similar to the results on the pressure series samples shown in Figure S2 of the Supporting Information. These peaks indicate the existence of glassy spins, [6, 7] which are frozen and do not contribute to the coercivity as much, below the temperature where coercivities reach their maxima. The peak locations also shift after the sample was gated. For the AG sample, the coercivity peak is located at 23.7 K, while the VC state peak is located at 19.8 K. This indicates that voltage conditioning can reduce spin frustration. A similar trend is also present in pressure series samples shown in Supporting Information Figure S2, that is, the temperatures where coercivities peak are larger for higher nitrogen concentration samples. For the recovered state, the coercivity does increase compared to the VC state, however, it didn't increase to the same level as the AG state, suggesting some voltage induced changes such as film structure changes are whichrreversible, whichh were also ”een ’n other magneto-ionic systems.[16-18]

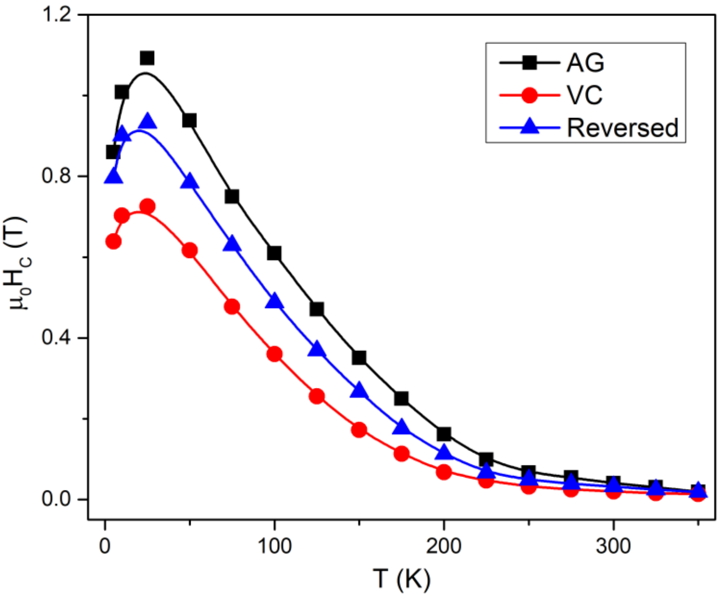

Figure S6: Temperature dependence of coercivity for the gating series. Error bars are smaller than the graph point size.

8. Polarized Neutron Reflectometry

Modeling of polarized neutron reflectometry (PNR) measurements benefits from a careful, step-wise approach in complexity due to solution degeneracy. Following this approach for the measurements performed on the POLREF instrument at ISIS Neutron and Muon Source at Rutherford Appleton Laboratory, we began by fitting data with the simplest models and then increased complexity, noting when additional parameters did not improve fit quality and when results were not consistent with physical parameters informed by other measurements (XRD, TEM, magnetometry, etc.). As a result, the "best" models chosen for the nitrogen, annealing, and gating series samples were the simplest models that yielded low χ^2 values and were consistent with the other characterization performed (profiles shown in main text Figure 5). Table S3 summarizes expected values for the nuclear component (ρ) of the scattering length density (SLD) for the potential phases of MnN_x and Ta(N and O_x).[19] For the chosen models discussed in the main text, tables S4-S6 contain the median fit values and 95% confidence intervals (Cis) for ρ and magnetic component of the SLD (ρ_M).

Table S3. Expected ρ Values

Material	ρ (× 10^{-6} Å^{-2})
Mn	-3.013
Mn_4N	-1.055
Mn_2N	0.632
Mn_3N_2	1.376
Ta	3.830
TaN	6.885
Ta_2O_5	4.787

Table S4. Pressure Series PNR Fit Values

P_N	Layer Material	ρ (× 10^{-6} Å^{-2}) [*95% CI*]	ρ_M (× 10^{-6} Å^{-2}) [*95% CI*]
**0%**			
	MnN_x-I	-0.869 [*-0.952 – -0.781*]*	0.045 [*0.002 – 0.110*]
	MnN_x-II	-1.493 [*-1.540 – -1.451*]†	0.235 [*0.203 – 0.273*]
	Ta	5.441 [*3.801 – 6.930*]	-
**2%**			
	MnN_x-I	-0.545 [*-0.589 – -0.497*]*	0.085 [*0.016 – 0.147*]
	MnN_x-II	-1.089 [*-1.116 – -1.062*]†	0.293 [*0.263 – 0.325*]‡
	Ta	4.688 [*3.735 – 6.178*]	-
**6%**			
	MnN_x-I	0.294 [*0.262 – 0.327*]*	0.062 [*0.031 – 0.090*]
	MnN_x-II	-0.540 [*-0.562 – -0.518*]†	0.218 [*0.199 – 0.241*]‡
	Ta	6.308 [*4.957 – 6.983*]	-

*, †, ‡ indicate the sample states which have no overlap in their 95% Cis.

Table S5. Annealing Series PNR Fit Values

Anneal Temperature	Layer Material	ρ (× 10^{-6} Å^{-2}) [*95% CI*]	ρ_M (× 10^{-6} Å^{-2}) [*95% CI*]
**Ref**			
	MnN_x-I	0.472 [*0.324 – 0.624*]*	0.097 [*0.085 – 0.100*]
	MnN_x-II	-0.809 [*-1.086 – -0.593*]	0.272 [*0.236 – 0.316*]
	TaN	3.671 [*3.610 – 3.748*]†	-
	Ta	3.443 [*3.367 – 3.521*]§	-
	TaO_x	4.817 [*3.142 – 5.955*]	-
**700 K**			
	MnN_x-I	-0.111 [*-0.230 – 0.130*]*	0.089 [*0.051 – 0.100*]
	MnN_x-II	-0.552 [*-0.628 – -0.486*]	0.289 [*0.255 – 0.326*]
	TaN	3.735 [*3.676 – 3.793*]‡	-
	Ta	3.497 [*3.427 – 3.532*]	-
	TaO_x	4.716 [*3.737 – 5.908*]	-
**750 K**			
	MnN_x-I	-0.384 [*-0.496 – -0.254*]*	0.075 [*0.020 – 0.100*]
	MnN_x-II	-0.736 [*-0.783 – -0.684*]	0.281 [*0.250 – 0.311*]
	TaN	4.033 [*3.962 – 4.141*]†,‡	-
	Ta	3.580 [*3.527 – 3.615*]§	-
	TaO_x	3.952 [*3.779 – 4.305*]	-

*, †, ‡, § indicate the sample states which have no overlap in their 95% Cis.

Table S6. Gating Series PNR Fit Values

Gating State	Layer Material	ρ (× 10^{-6} Å^{-2}) [*95% CI*]	ρ_M (× 10^{-6} Å^{-2}) [*95% CI*]
**As Grown**			
	MnN_x-I	0.120 [*-0.012 – 0.267*]	0.210 [*0.119 – 0.338*]
	MnN_x-II	-0.717 [*-0.810 – -0.632*]*	0.335 [*0.255 – 0.408*]
	Ta	3.538 [*3.504 – 3.569*]†	-
	TaO_x	5.143 [*4.127 – 5.973*]	-
**VC**			
	MnN_x-I	0.168 [*0.013 – 0.355*]	0.153 [*0.070 – 0.241*]
	MnN_x-II	-0.575 [*-0.663 – -0.495*]*	0.258 [*0.196 – 0.322*]
	Ta	3.707 [*3.674 – 3.746*]†	-
	TaO_x	4.865 [*3.782 – 5.944*]	-

* indicates sample states that have overlap in 95% CI between them, but calculated statistical probability of no change between states < 0.05

† indicates sample states that have no overlap in 95% CI between them

The fitting process and support for the chosen model are further explained in the following subsections by highlighting under and over parameterized models for each sample series. Additionally, the fit data for the models that were chosen are provided, with further detail on sample-to-sample variations in each series. It should be noted that only a small subset of the models that were used during the fitting process are shown here, and many more possible solutions were explored.

8.1. Pressure Series PNR

The fitted reflectivity and spin asymmetry $(R^+ - R^-)/(R^+ + R^-)$ for the chosen models for the pressure series samples (main text Figure 5a) are shown below in Figure S7. The theoretical fits (shown as solid lines) have good agreement with the reflectivity for each sample in the series, with χ^2 between 1.65 and 2.10. The spin asymmetry that arises from the magnetic contributions to the SLD is also well captured by the theoretical fits, as shown in Figure S7d-f. Qualitatively, notable changes in the overall reflectivity and spin asymmetry are observed, especially at lower values of Q. These changes are primarily manifested in the resulting SLD profiles (Figure 5a) as ρ increases in MnN_x and slight variations in magnetization with increasing nitrogen pressure.

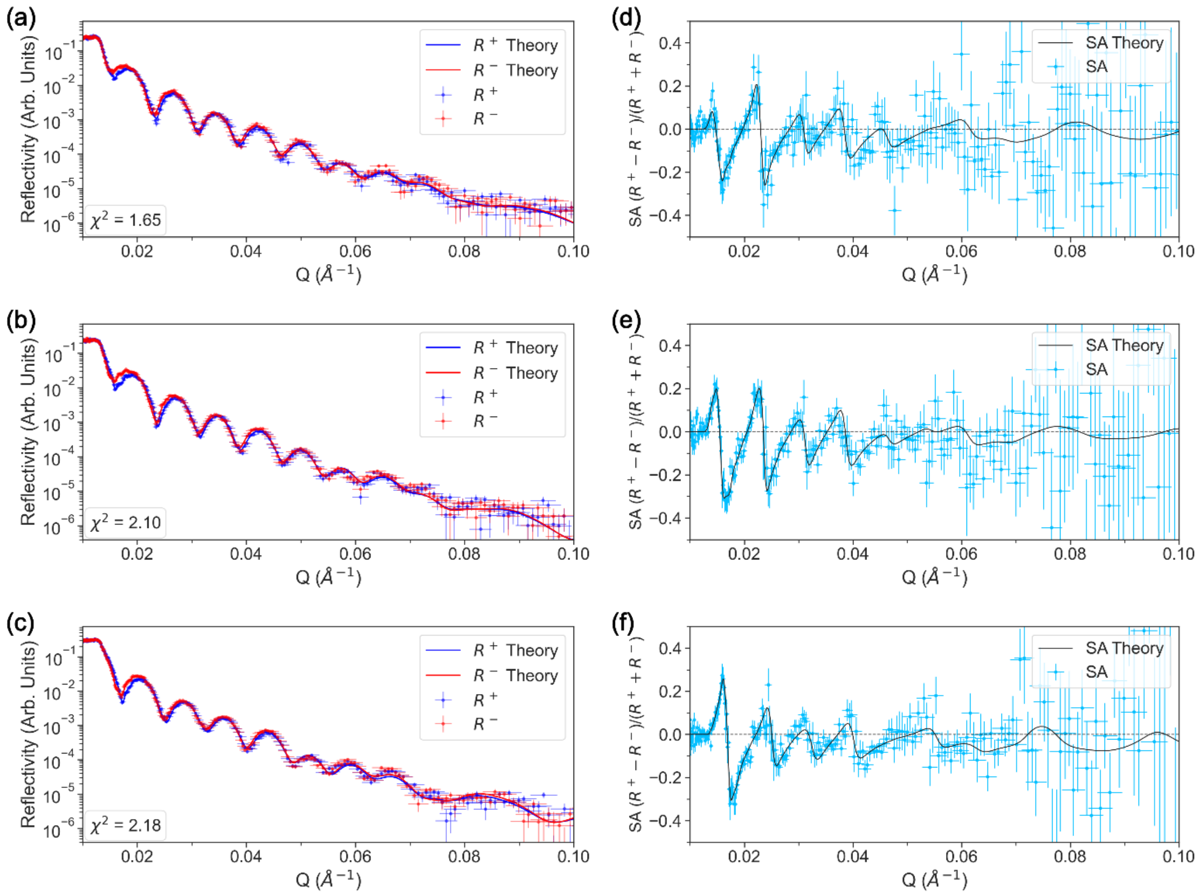

Figure S7: Polarized neutron reflectivity for the pressure series samples. Reflectivity fits (a-c) and calculated spin asymmetry (d-f), SA, for the P_N = 0% (a, d), P_N = 2% (b, e), and P_N = 6% (c, f) samples. For reflectivity, the reduced data are shown as points with reflectivity and Q error bars, and the theoretical fits are shown as solid lines with a corresponding color to the R^+ or R^- cross section. The SA calculated from the data is shown as points with SA and Q error bars, and the theoretically calculated SA is shown as a black solid line.

Examples of less complex models used for the pressure series are shown in Figure S8. A "bulk" model used for P_N = 0% is shown in Figure S8a-c, where MnN_x is treated as a single layer with constant SLDs. Compared to the chosen model, this bulk model has a higher χ^2, the theotical reflectivity does not agree well with the data for Q > 0.05 Å^{-1}, and a slight phase shift is aparent in the spin asymmetry. For another model, which is of similar complexity to the chosen model, the MnN_x layers were inverted (Figure S8 d-f). Again, the higher χ^2, worse theoretic agreement in the reflectivity at Q > 0.05 Å^{-1}, and a phase shift in the spin asymmetry led to the exclusion of this model. Additionally, the poorer fit from this model supports the stacking order of the layers in the chosen model. Similar, less complex models were used for the P_N = 2% and P_N = 6% samples, with similar trends in fit quality as shown here. Two examples of more complex models that were used during the fitting process for the P_N = 0% sample are shown in Figure S9. The first model, shown in Figure S9a-c has comparable parameters to the chosen model for all layers, except that Ta was split into two layer (Ta and

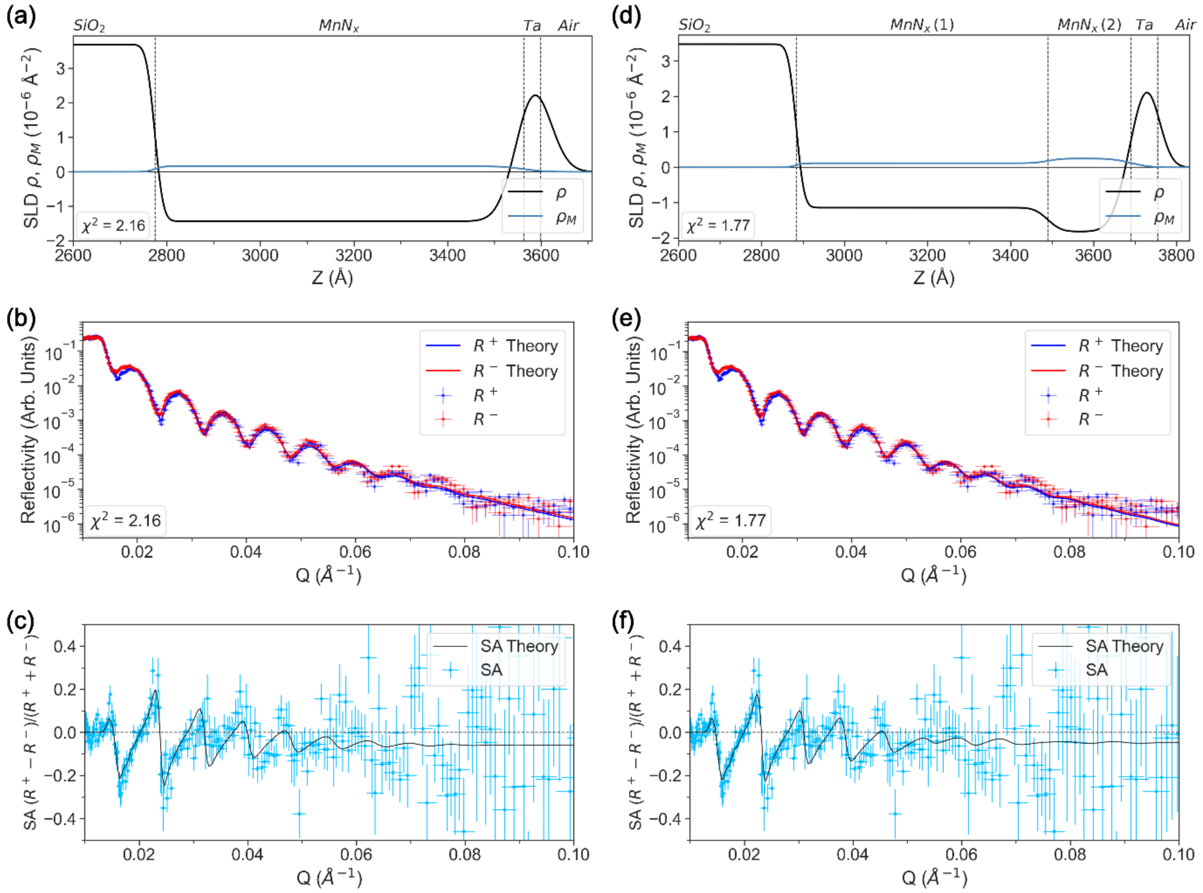

Figure S8: Pressure series PNR of less complex models. The SLD profile, reflectivity fits, and spin asymmetry for (a-c) a "bulk" model with constant SLD values for the entire MnN_x thickness and (d-f) a model with thicknesses in MnN_x sublayers that are inverted.

TaO_x). No improvements over the chosen model were noted for χ^2 or the overall fit for reflectivity or spin asymmetry, so this model was considered over parameterized and discarded. The second model shown in Figure S9d-f had similar parameters to the chosen model, except the MnN_x layer was split into three layers instead of two. Even when allowing various ranges for the thicknesses and SLDs for these three MnN_x layers, no significant improvement in χ^2 or overall fit was achieved, so this model was also discarded as it is over parameterized. These over parameterized models, among others that were attempted during the fitting process, also did not lead to any improvemetns over the chosen models for the other samples in the pressure series.

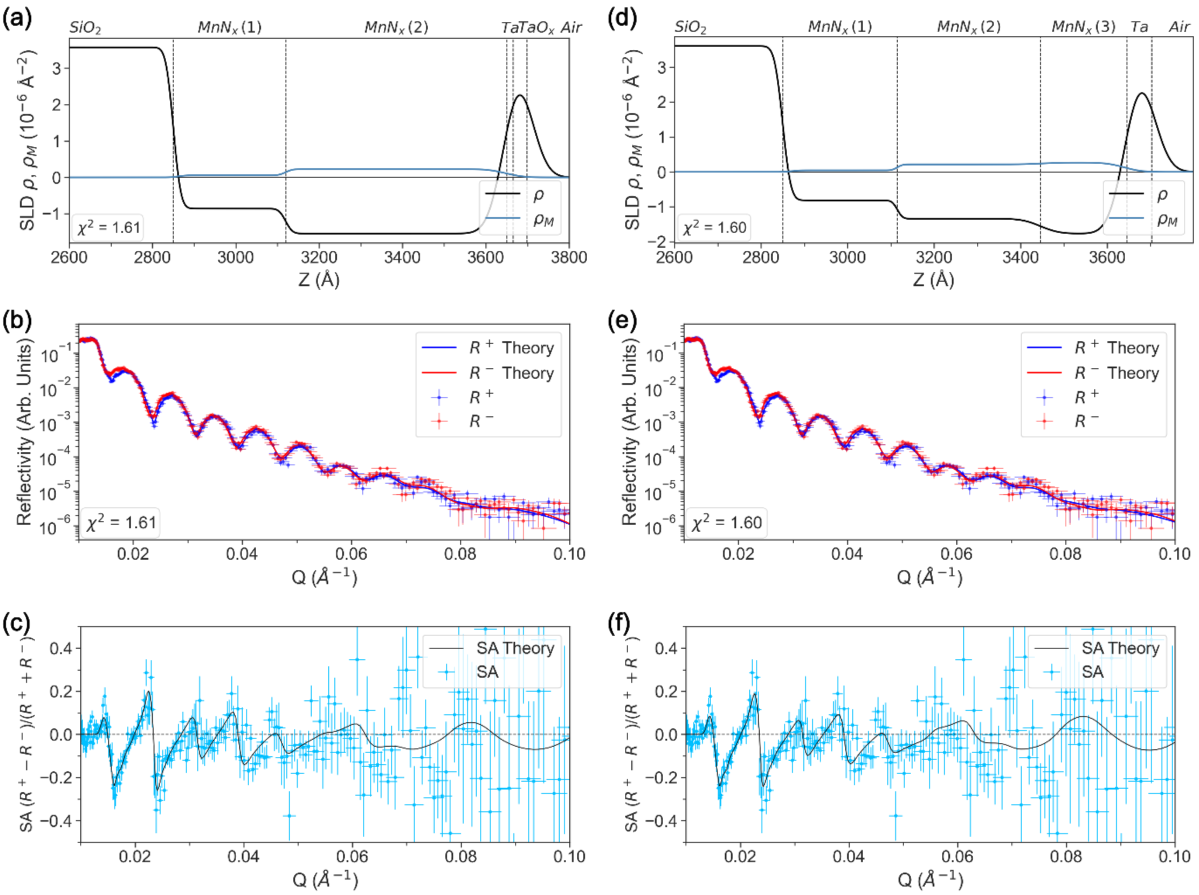

Figure S9: Pressure series PNR of more complex models. Models that are more complex compared to the main text chosen model. The profiles, reflectivity, and spin asymmetry are shown for (a-c) a model with TaO_x included as part of the top Ta layer, and (d-f) a model that includes three MnN_x layers.

8.2 Annealing Series PNR

The reflectivity and spin asymmetry fits for the annealing series samples’ chosen models (main text Figure 5b) are shown in Figure S10. Theoretical fits from the models had resulting χ^2 between 1.58 and 1.71, and both the theoretical reflectivity and spin asymmetry are in qualitative agreement with the reduced data. Between each sample in the annealing series, difference in reflectivity are more aparrent across the entire Q range compared to the pressure series samples. Yet, Figure 5b shows SLD changes in the MnN_x layer that are similar in magnetude as the pressure series samples. Due to similarities in individual and combined layer thicknesses between the TaO_x/Ta/TaN layers and the MnN_x layers, these more apparent changes in reflectivity likely arise from the dramatic effects small changes in SLD or thickness can have on superimposed reflectivity fringes that are close in periodicity (or multiples of periodicity).

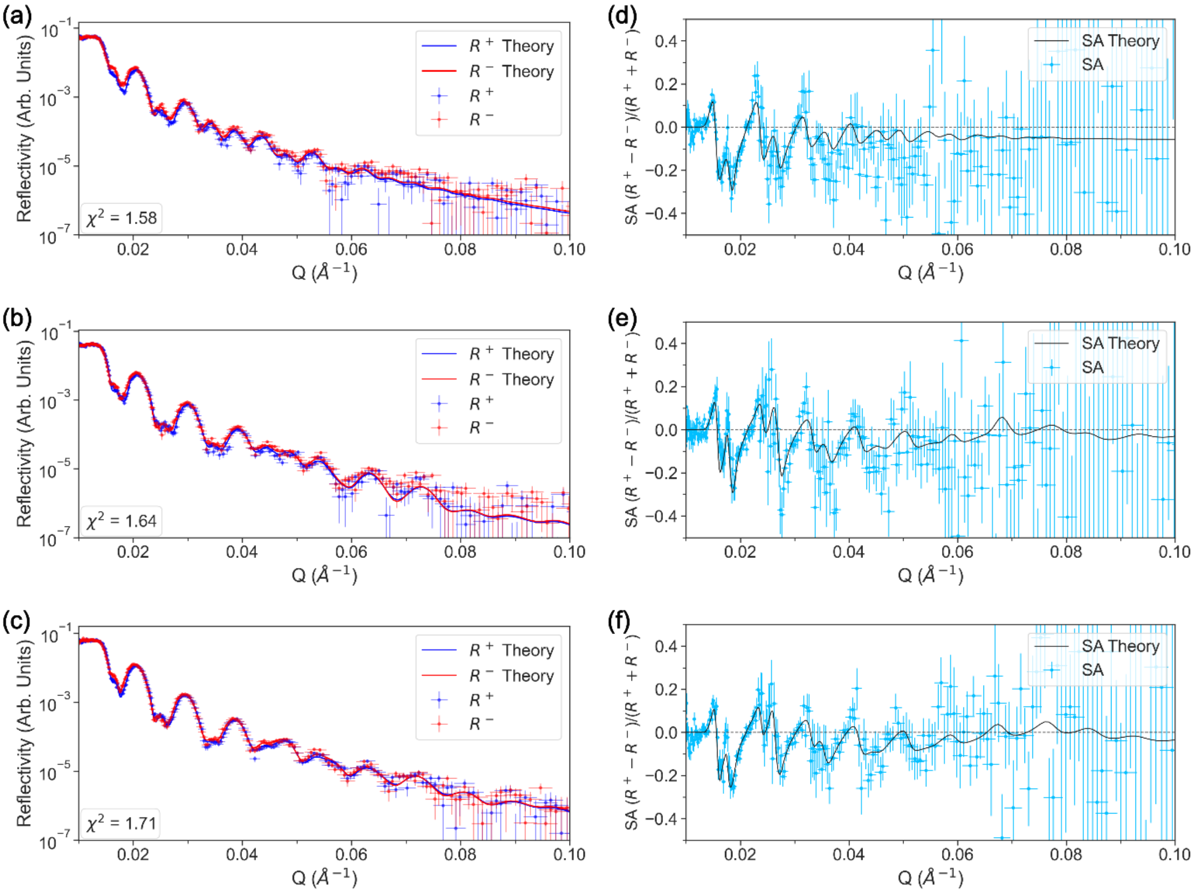

Figure S10: PNR for the annealed series samples. Reflectivity fits (a-c) and calculated spin asymmetry (d-f), SA, for the AG (a, d), 700 K (b, e), and 750 K (c, f) sample states. For reflectivity, the reduced data are shown as points with reflectivity and Q error bars, and the theoretical fits are shown as solid lines with a corresponding color to the R+ or R- cross section. The SA calculated from the data is shown as points with SA and Q error bars, and the theoretically calculated SA is shown as a black solid line.

Several models that were less complex compared to the chosen annealing series as-grown (AG) model are shown in Figure S11. The first is a model where bulk values for SLD were used for a single MnN_x layer and Ta layer (Figure S11a-c). This model was quickly dismissed as it has a worse χ^2 and the theoretical reflectivity deviates from the reduced data for large sections of Q throughout the entire range. In Figure S11d-f, a very similar model to that chosen is shown, except that the Ta layer is not broken in to TaN and TaO_x sub-layers. There is a slightly higher χ^2 in this model and small phase shifts in the overall reflectivity and spin asymmetry between Q of 0.015 Å^{-1} and 0.030 Å^{-1}. We believe the improvement in the fit produced by included additional sub-layers of TaN and TaO_x in the Ta layer for the chosen model is justified by the quantitative improvement in χ^2 along with the qaulitative improvement in the fits at lower values of Q. Additionally, these same simpler models were used on the samples annealed at 700 K and 750 K (not shown), where the quality of the fits became worse with increasing temperature while the chosen models, all with the same layer structure, produced good fits for all samples in the series.

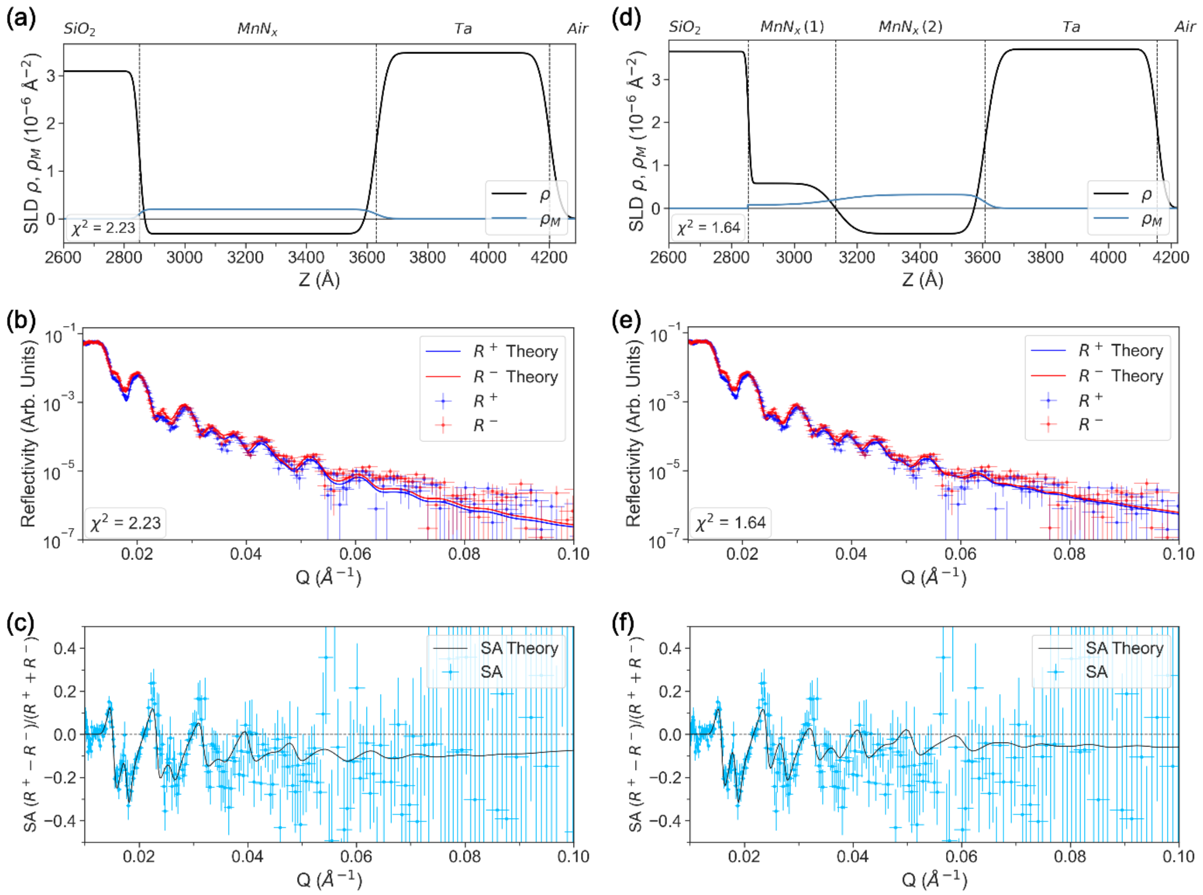

Figure S11: PNR of less complex models for the annealing series. SLD profiles, fit reflectivity, and spin asymmetry for (a-c) a simple "bulk" model where MnN_x and Ta layers are modeled with SLD values equal across their thickness, and (d-f) a model with comparable fit parameters to the chosen model, except Ta is treated as a uniform layer.

Examples of more complex models that were used for the annealed series 700 K and 750 K fitting are shown in Figure S12. Here, Figure S12a-c shows a model for the 700 K sample, where all parameters were similar to the chosen model, except the magnetic SLD profile was allowed to be include a magnetic dead-layer within the MnN_x (1) layer. This model offers no significant improvement in χ^2 or qualitative fit of the reflectivity or spin asymmetry, so it was dismissed as over parameterized. The second model shown in Figure S12d-f, for the 750 K sample, shows another example of adding additional layers to the MnN_x. This again did not improve the overall fits and was dismissed. Similar models with addition layers, varying contraints, and linking/unlinking parameters were used on all samples in the annealing series, none of which offered a significant enough improvement to justify them over the chosen model presented in the main text.

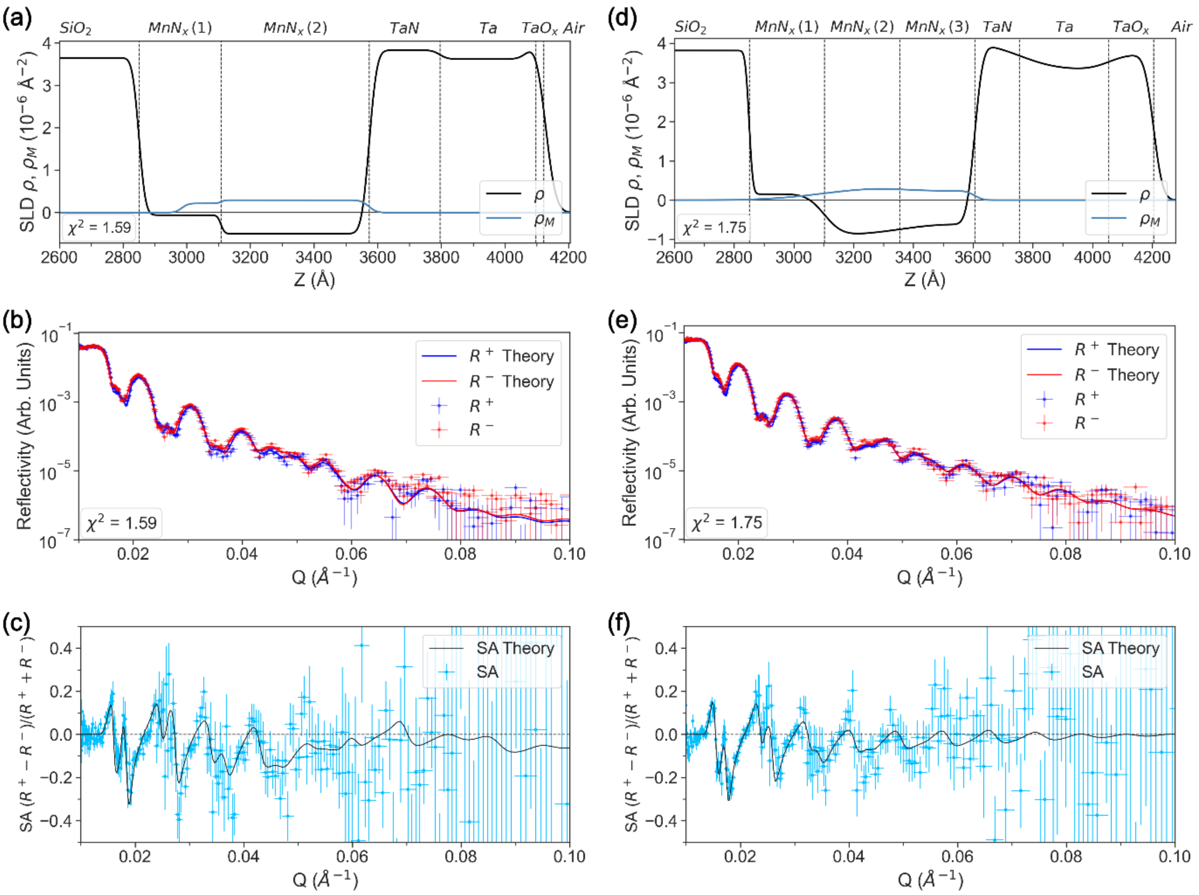

Figure S12: PNR of more complex models used for the annealing series samples. Models that are more complex compared to the main text chosen model. The profiles, reflectivity, and spin asymmetry are shown for (a-c) a model allowing a large magnetic dead layer withing the MnN_x (1) layer, and (d-f) a model that includes three MnN_x layers.

8.3 Gating Series PNR

Fitting of gating series samples varied slightly from the other two series samples. First, a smaller area for the samples was required for gating (25 mm^2 vs. 100-400 mm^2 for other series), and this size reduction significantly increases the counting time required to reach similar statistics and can have effects on the resolution. Additionally, the samples were grown and cleaved such that there was a roughly 1-2 mm SiO_2/Si boarder around the 5 mm × 5 mm films. Since both the film and boarder contribute to the reflectivity, fitting was performed using a mixed model of a full sample stack and a "blank" sample. The sample fractional area ($\frac{A_{film}}{A_{film}+A_{boarder}}$) between the two was a fitting parameter, and was estimated to be between 0.4 and 0.6 for each sample by manual measurement. This is in agreement with the fitted value for sample fractional area for each of the chosen models.

The fit reflectivity and spin aymmetry for the gating series samples are shown in Figure S13, which corresponds to the SLD profiles shown in the main text Figure 5c. Overall

reflectivity, spin asymmetry, and χ^2 suggest good fits using these models. A qualitative inspection of the reflectivity does not reveal any notable differences between the two states, and spin asymmetry only shows slight variations that may be within the level of uncertainty. Still, there were statistically significant differences between the two states models that suggest N is moving "upward" in the samples (discussed further in main text).

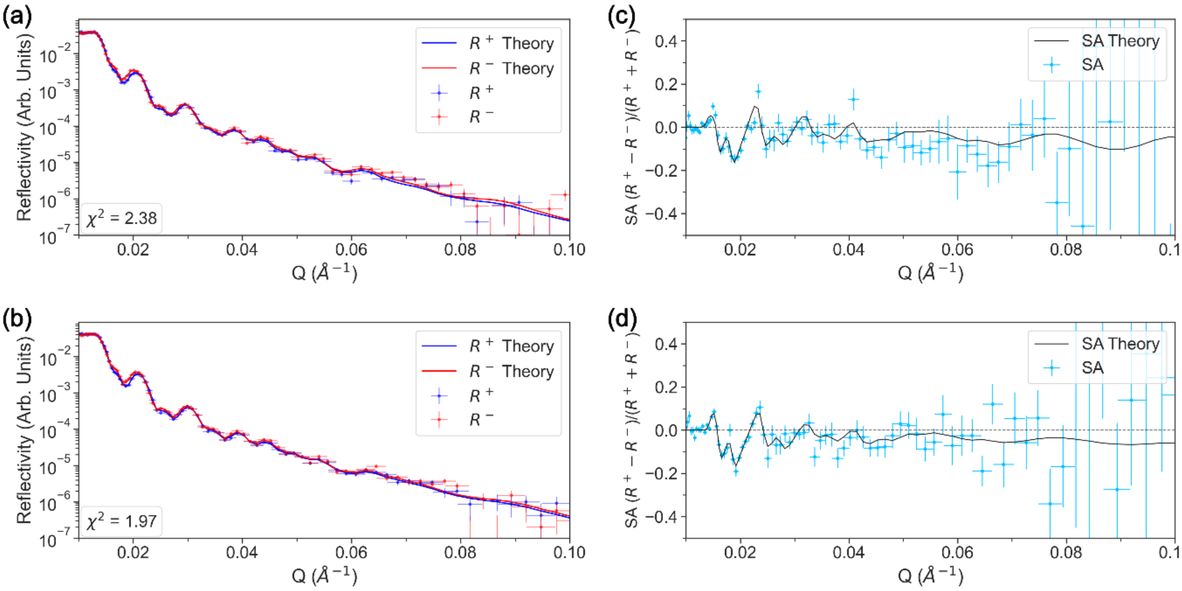

Figure S13: PNR for the gating series samples. Reflectivity and theoretical fits (a-b) and spin asymmetry (c-d) for the (a,c) AG state and (b, d) after gating state of the voltage series samples. For reflectivity, the reduced data are shown as points with reflectivity and Q error bars, and the theoretical fits are shown as solid lines with a corresponding color to the R+ or R- cross section. The SA calculated from the data is shown as points with SA and Q error bars, and the theoretically calculated SA is shown as a black solid line.

Again, simpler and more complex models were used while working toward the best model for this series of samples. Since the gating series samples were grown with the same parameters as the annealed series AG sample, similar models were attempted. A full discussion of figures of alternate fits is omitted here since the discussion largely follows that of the previous annealed series section. The only difference from the annealed series is the exclusion of a TaN sub-layer, which could not be justified from the differences in fit quality. It could be that the samples are truly different, or the reduced statistics on the gating series samples was not enough to resolve another sub-layer in Ta.

References

(1) Chen, Z.; Jensen, C. J.; Liu, C.; Zhang, X.; Liu, K. Ionically Driven Synthesis and Exchange Bias in Mn_4N/MnN_x Heterostructures. *Appl. Phys. Lett.* **2023**, *123*, 082403. DOI: 10.1063/5.0165895.

(2) Li, W.; Xu, X.; Gao, T.; Harumoto, T.; Nakamura, Y.; Shi, J. Enhanced perpendicular magnetic anisotropy of ferrimagnetic Mn_4N films deposited on the glass substrate. *J. Phys. D: Appl. Phys* **2022**, *55*, 275004. DOI: 10.1088/1361-6463/ac5e1b.

(3) Ding, J. F.; Lebedev, O. I.; Turner, S.; Tian, Y. F.; Hu, W. J.; Seo, J. W.; Panagopoulos, C.; Prellier, W.; Van Tendeloo, G.; Wu, T. Interfacial Spin Glass State and Exchange Bias in Manganite Bilayers with Competing Magnetic Orders. *Phys. Rev. B* **2013**, *87*, 054428. DOI: 10.1103/PhysRevB.87.054428.

(4) Wang, C.; Zhou, L.; Fu, Q.; Tian, Y.; Wang, S.; Gou, H.; Ai, J.; Zhang, L.; Xue, F. Exchange Bias in Spin-Glass-Like $NiFe_2O_4/BiFeO_3$ Heterojunction at Room Temperature. *J. Magn. Magn. Mater.* **2018**, *449*, 372-377. DOI: 10.1016/j.jmmm.2017.10.054.

(5) Nayak, S.; Manna, P. K.; Singh, B. B.; Bedanta, S. Effect of Spin Glass Frustration on Exchange Bias in NiMn/CoFeB Bilayers. *Phys. Chem. Chem. Phys.* **2021**, *23*, 6481. DOI: 10.1039/d0cp05726f.

(6) Ali, M.; Adie, P.; Marrows, C. H.; Greig, D.; Hickey, B. J.; Stamps, R. L. Exchange Bias Using a Spin Glass. *Nat. Mater.* **2007**, *6*, 70-75. DOI: 10.1038/nmat1809.

(7) Del Bianco, L.; Fiorani, D.; Testa, A.; Bonetti, E.; Signorini, L. Field-cooling dependence of exchange bias in a granular system of Fe nanoparticles embedded in an Fe oxide matrix. *Phys. Rev. B* **2004**, *70*, 052401. DOI: 10.1103/PhysRevB.70.052401.

(8) Karmakar, S.; Taran, S.; Bose, E.; Chaudhuri, B. K.; Sun, C. P.; Huang, C. L.; Yang, H. D. Evidence of Intrinsic Exchange Bias and Its Origin in Spin-Glass-Like Disordered $L_{0.5}Sr_{0.5}MnO_3$ Manganites (L=Y,$Y_{0.5}Sm_{0.5}$, and $Y_{0.5}La_{0.5}$). *Phys. Rev. B* **2008**, *77*, 144409. DOI: 10.1103/PhysRevB.77.144409.

(9) Rui, W. B.; Hu, Y.; Du, A.; You, B.; Xiao, M. W.; Zhang, W.; Zhou, S. M.; Du, J. Cooling Field and Temperature Dependent Exchange Bias in Spin Glass/Ferromagnet Bilayers. *Sci. Rep.* **2015**, *5*, 13640. DOI: 10.1038/srep13640.

(10) Giri, S. K.; Poddar, A.; Nath, T. K. Evidence of Exchange Bias Effect and Surface Spin Glass Ordering in Electron Doped $Sm_{0.09}Ca_{0.91}MnO_3$ Nanomanganites. *J. Appl. Phys.* **2012**, *112*, 113903. DOI: 10.1063/1.4767926.

(11) Zhang, J.; Yang, J.; Causer, G. L.; Shi, J.; Klose, F.; Huang, J.-K.; Tseng, A.; Wang, D.; Zu, X.; Qiao, L.; Pham, A.; Li, S. Realization of Exchange Bias Control with Manipulation of Interfacial Frustration in Magnetic Complex Oxide Heterostructures. *Phys. Rev. B* **2021**, *104*, 174444. DOI: 10.1103/PhysRevB.104.174444.

(12) Gilbert, D. A.; Liao, J.-W.; Wang, L.-W.; Lau, J. W.; Klemmer, T. J.; Thiele, J.-U.; Lai, C.-H.; Liu, K. Probing the *A*1 to $L1_0$ transformation in FeCuPt using the first order reversal curve method. *APL Mater.* **2014**, *2*, 086106. DOI: 10.1063/1.4894197.

(13) Burks, E. C.; Gilbert, D. A.; Murray, P. D.; Flores, C.; Felter, T. E.; Charnvanichborikarn, S.; Kucheyev, S. O.; Colvin, J. D.; Yin, G.; Liu, K. 3D Nanomagnetism in Low Density Interconnected Nanowire Networks. *Nano Lett.* **2021**, *21*, 716−722. DOI: 10.1021/acs.nanolett.0c04366.

(14) Gilbert, D. A.; Zimanyi, G. T.; Dumas, R. K.; Winklhofer, M.; Gomez, A.; Eibagi, N.; Vicent, J. L.; Liu, K. Quantitative Decoding of Interactions in Tunable Nanomagnet Arrays Using First Order Reversal Curves. *Sci. Rep.* **2014**, *4*, 4204. DOI: 10.1038/srep04204.

(15) Kou, X.; Fan, X.; Dumas, R. K.; Lu, Q.; Zhang, Y.; Zhu, H.; Zhang, X.; Liu, K.; Xiao, J. Q. Memory Effect in Magnetic Nanowire Arrays. *Adv. Mater.* **2011**, *23*, 1393-1397. DOI: 10.1002/adma.201003749.

(16) Gilbert, D. A.; Grutter, A. J.; Arenholz, E.; Liu, K.; Kirby, B. J.; Borchers, J. A.; Maranville, B. B. Structural and Magnetic Depth Profiles of Magneto-Ionic Heterostructures beyond the Interface Limit. *Nat. Commun.* **2016**, *7*, 12264. DOI: 10.1038/ncomms12264.

(17) de Rojas, J.; Quintana, A.; Rius, G.; Stefani, C.; Domingo, N.; Costa-Krämer, J. L.; Menéndez, E.; Sort, J. Voltage Control of Magnetism with Magneto-Ionic Approaches: Beyond Voltage-Driven Oxygen Ion Migration. *Appl. Phys. Lett.* **2022**, *120*, 070501. DOI: 10.1063/5.0079762.

(18) Kossak, A. E.; Kaczmarek, A. C.; Valvidares, M.; Gargiani, P.; Hasan, M. U.; Beach, G. S. D. Ionic Modulation of Ferromagnetic and Proximity-Induced Magnetization in Co/Pd Heterostructures. *Adv. Funct. Mater.* **2024**, 2403858. DOI: 10.1002/adfm.202403858.

(19) Kienzle, P. Neutron activation and scattering calculator, NIST Center for Neutron Research, revised March 25, 2024. https://www.ncnr.nist.gov/resources/activation/.
